# Substrate-Assisted Catalysis in Polyketide Reduction Proceeds via a Phenolate Intermediate

**DOI:** 10.1016/j.chembiol.2016.07.018

**Published:** 2016-09-22

**Authors:** Martin Schäfer, Clare E.M. Stevenson, Barrie Wilkinson, David M. Lawson, Mark J. Buttner

**Affiliations:** 1 Department of Molecular Microbiology, John Innes Centre, Norwich Research Park, Norwich NR4 7UH, UK; 2 Department of Biological Chemistry, John Innes Centre, Norwich Research Park, Norwich NR4 7UH, UK

## Abstract

SimC7 is a polyketide ketoreductase involved in biosynthesis of the angucyclinone moiety of the gyrase inhibitor simocyclinone D8 (SD8). SimC7, which belongs to the short-chain dehydrogenase/reductase (SDR) superfamily, catalyzes reduction of the C-7 carbonyl of the angucyclinone, and the resulting hydroxyl is essential for antibiotic activity. SimC7 shares little sequence similarity with characterized ketoreductases, suggesting it might have a distinct mechanism. To investigate this possibility, we determined the structures of SimC7 alone, with NADP^+^, and with NADP^+^ and the substrate 7-oxo-SD8. These structures show that SimC7 is distinct from previously characterized polyketide ketoreductases, lacking the conserved catalytic triad, including the active-site tyrosine that acts as central acid-base catalyst in canonical SDR proteins. Taken together with functional analyses of active-site mutants, our data suggest that SimC7 catalyzes a substrate-assisted, two-step reaction for reduction of the C-7 carbonyl group involving intramolecular transfer of a substrate-derived proton to generate a phenolate intermediate.

## Highlights

• SimC7 catalyzes reduction of the C-7 carbonyl of the angucyclinone of simocyclinone
• SimC7 lacks the conserved catalytic triad characteristic of canonical ketoreductases
• SimC7 reduces the C-7 carbonyl via a substrate-assisted, two-step reaction
• This involves intramolecular transfer of a substrate proton to generate a phenolate

## Graphical Abstract

**Figure:**
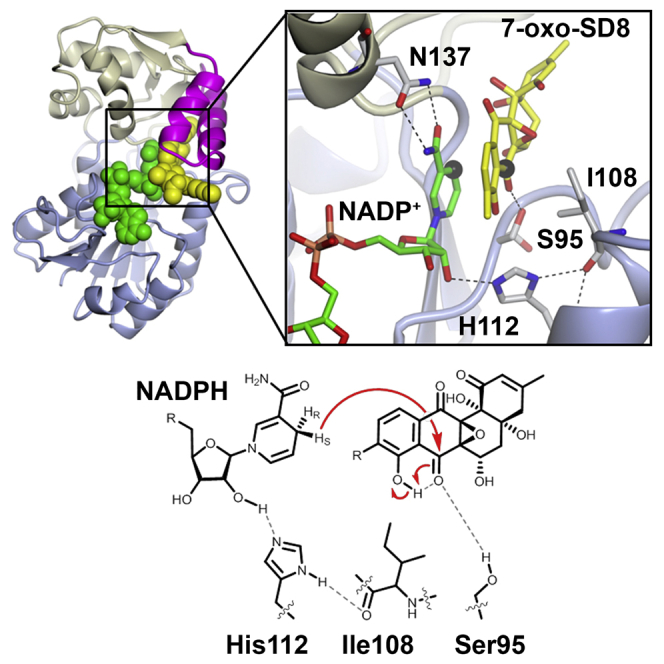

## Teaser

SimC7 is a polyketide ketoreductase involved in the biosynthesis of the angucyclinone moiety of the DNA gyrase inhibitor simocyclinone. It is structurally distinct from previously characterized ketoreductases, lacking the canonical Ser-Tyr-Lys catalytic triad. Instead, SimC7 catalyzes a substrate-assisted, two-step reaction involving an unusual phenolate intermediate.

## Introduction

Angucyclin(on)es form the largest group of polycyclic aromatic polyketides, many with anticancer and antibacterial activities (Kharel et al., 2012). They share a polyketide-derived tetracyclic benz[*a*]anthracene carbon skeleton, but numerous structures are generated by a range of tailoring reactions, including O- or C-linked deoxysugar glycosylation to form angucyclines. In general, these tailoring enzymes are not well understood. An important challenge, therefore, is to define the step catalyzed by each enzyme and to determine their reaction mechanisms. This knowledge is particularly relevant to the rational engineering of angucyclin(on)e biosynthetic pathways for novel therapeutics.

**Figure 1:**
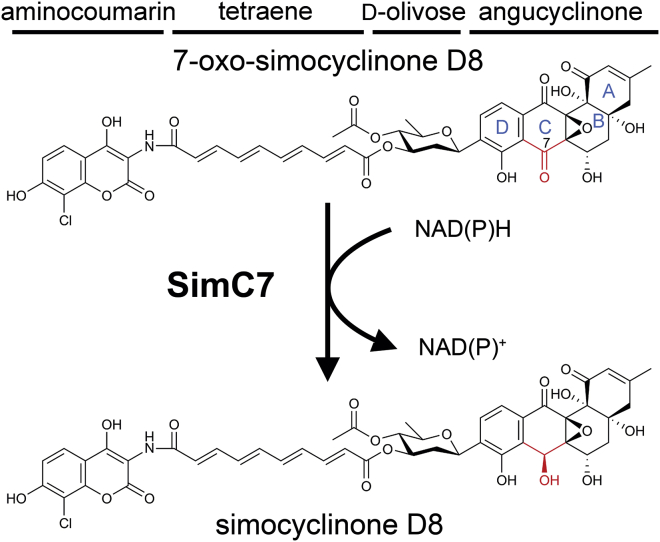
SimC7 Catalyzes the Reduction of 7-oxo-SD8 to Simocyclinone D8 A, B, C, and D denote the four rings of the angucyclinone moiety; the C-7 carbonyl/hydroxyl is highlighted in red.

Simocyclinone D8 (SD8) is a potent DNA gyrase inhibitor isolated from *Streptomyces antibioticus* that consists of an angucyclinone connected to a chlorinated aminocoumarin via a D-olivose deoxysugar and a tetraene diester linker (Figure 1) (Schimana et al., 2000, Edwards et al., 2009, Hearnshaw et al., 2014). SD8 is bifunctional, with the angucyclinone and the aminocoumarin at opposite ends of the molecule binding to two distinct pockets on the DNA binding surface of the GyrA subunit of gyrase (Edwards et al., 2009, Hearnshaw et al., 2014), thereby inhibiting DNA supercoiling at submicromolar concentrations (Edwards et al., 2011). Because gyrase is essential in bacteria but absent from humans, it is an attractive target for antimicrobial drugs, as exemplified by the clinically successful fluoroquinolones (Collin et al., 2011).

SimC7 was originally annotated as a dehydratase and predicted to be involved in the biosynthesis of the tetraene linker of SD8 (Trefzer et al., 2002). However, we recently showed that SimC7 is in fact an NAD(P)H-dependent ketoreductase that catalyzes the reduction of a carbonyl to a hydroxyl group at the C-7 position of the angucyclinone (Schäfer et al., 2015). This enzymatic step is essential for antibiotic activity, converting the almost inactive 7-oxo-simocyclinone D8 (7-oxo-SD8; half maximal inhibitory concentration [IC_50_] ∼50–100 μM) into the potent gyrase inhibitor SD8 (IC_50_ ∼0.1–0.6 μM) (Schäfer et al., 2015).

Based on the intermediates produced by *S. antibioticus*, it seems that the biosynthesis of SD8 starts with assembly of the angucyclinone, followed by the attachment of the deoxysugar, then the tetraene linker, and finally the aminocoumarin (i.e., SD8 is assembled from right to left in Figure 1) (Schimana et al., 2001). Therefore, the physiological substrate of SimC7 is most likely a 7-oxo angucyclinone intermediate lacking the attached deoxysugar, tetraene linker, and aminocoumarin, an intermediate that is detectable only in *ΔsimC7* mutants (Schäfer et al., 2015). Despite this, the enzyme readily accepts as a substrate the full-length intermediate 7-oxo-SD8, the major product made by *ΔsimC7* mutants (Schäfer et al., 2015).

The angucyclinone moiety of SD8 is synthesized by a type II polyketide synthase (SimA1-3) and multiple tailoring enzymes (SimA4-13, SimC7) that catalyze cyclization, aromatization, oxidation, and reduction reactions. Several ketoreductases of the short-chain dehydrogenase/reductase (SDR) family that act on angucyclinones or related polyketides have been characterized. The reduction of carbonyl groups at the C-6 and C-9 positions of polyketides has been functionally characterized, and the structures of the corresponding SDR enzymes have elucidated their reaction mechanisms and factors determining their stereoselectivity. The ketoreductases LanV and UrdMred act on the C-6 carbonyl group of angucyclic polyketides from the landomycin and urdamycin pathways (Paananen et al., 2013, Patrikainen et al., 2014). In contrast, the ketoreductases ActKR and HedKR act on the C-9 carbonyl group of early intermediates in the actinorhodin and hedamycin polyketide pathways (Javidpour et al., 2011a, Javidpour et al., 2011b, Javidpour et al., 2013, Korman et al., 2004, Korman et al., 2008). The LanV, UrdMred, ActKR, and HedKR structures revealed the catalytic Ser-Tyr-Lys triad characteristic of SDR enzymes, where the latter two residues form a YxxxK motif. In these classical SDR proteins, the conserved active-site tyrosine serves as central acid-base catalyst that donates a proton to the substrate. The adjacent lysine residue lowers the pK_a_ of the tyrosine hydroxyl group and often contributes directly to a proton relay mechanism, and the hydroxyl group of the serine stabilizes and polarizes the carbonyl group of the substrate (Kavanagh et al., 2008).

At the sequence level, SimC7 shares little similarity with any characterized ketoreductase, even with functionally analogous polyketide ketoreductases. The striking differences between the amino acid sequence of SimC7 and those of HedKR, ActKR, LanV, and UrdMred suggested that SimC7 might have a novel catalytic mechanism. To investigate this possibility, we determined the structures of SimC7 alone (apo; 1.6 Å resolution), the binary complex with NADP^+^ (1.95 Å), and the ternary complex with both NADP^+^ and 7-oxo-SD8 (1.2 Å) (Tables S1 and S2). Our results reveal that SimC7 is structurally distinct from previously characterized polyketide ketoreductases and, importantly, lacks the canonical SDR Ser-Tyr-Lys catalytic triad (Kavanagh et al., 2008, Kallberg et al., 2010, Persson and Kallberg, 2013). Instead, our data suggest that SimC7 catalyzes a substrate-assisted, two-step reaction for the reduction of the C-7 carbonyl group involving an unusual phenolate intermediate.

## Results and Discussion

### Overall Structure of SimC7

**Figure 2:**
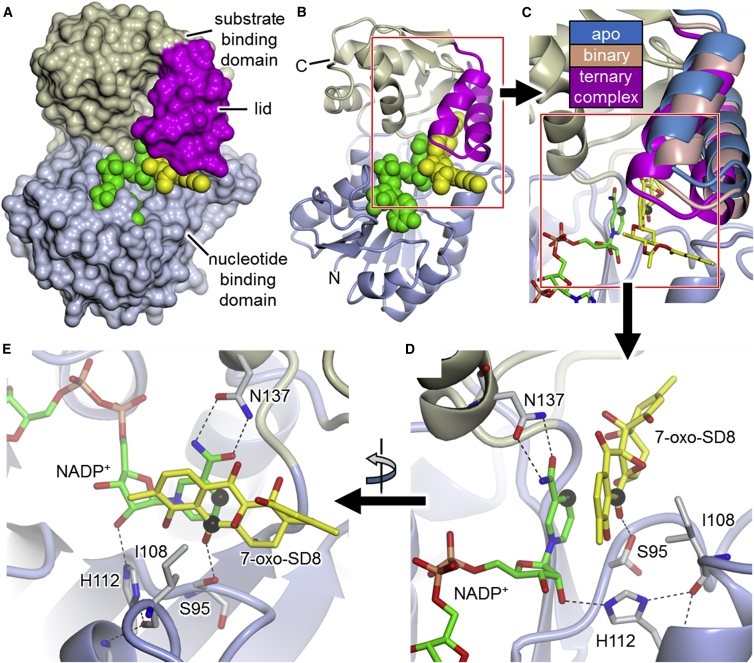
Crystal Structure of SimC7 (A and B) SimC7 displayed as (A) a molecular surface and (B) cartoon representation with the nucleotide binding domain, the substrate binding domain, and the lid motif shown in pale blue, beige, and magenta, respectively. Ligands are represented as van der Waals spheres with the 7-oxo-SD8 substrate shown in yellow and the cofactor shown in green. (C) Close-up showing conformational changes in the lid between the apo (chain A, form 2; blue), the binary complex (salmon), and the ternary complex (magenta); the core protein structure and ligands shown represent the ternary complex. C-4 of the cofactor and C-7 of the substrate are highlighted by black spheres, showing that C-7 of the substrate is exactly positioned 3 Å from C-4 of the nicotinamide ring, poised for direct hydride transfer. (D and E) Orthogonal close-ups showing the active site of the ternary complex including the Ser95-Ile108-His112 “catalytic triad” residues, and Asn137, which is important in maintaining the *syn* conformation of the cofactor. For clarity, only the angucyclic polyketide moiety of the substrate is shown. Hydrogen bonds are shown as dashed lines. (E) is also reproduced as a stereo image in Figures S3A and S3B, which also show a difference electron density map calculated from the final model after simulated annealing refinement with the substrate omitted. See also Figures S1–S4 and Tables S1–S3.

SimC7 is made up of two domains, the larger of which is the nucleotide binding domain that adopts a Rossmann fold (Figures 2A, 2B, and S1); the smaller substrate binding domain, characteristic of the so-called extended SDR subfamily (Kavanagh et al., 2008), is mainly α-helical and is largely formed by two insertions in the nucleotide binding domain (between β6 and α6, and between β9 and α10). Notably, the latter insertion contains a “lid” motif consisting of two antiparallel α helices (α8 and α9) that folds over the active site (Figures 2A, 2B, and S1). The substrate binding domain is completed by a short helical segment at the C-terminus of the polypeptide chain.

**Figure 3:**
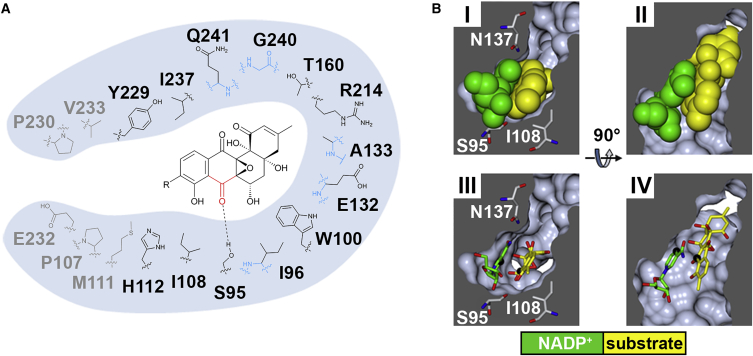
SimC7 Has a Very Hydrophobic and Constricted Substrate Binding Pocket (A) The substrate is bound by only one direct hydrogen bond between the C-7 carbonyl group (red) in the angucyclinone moiety and the side-chain hydroxyl of Ser95. This interaction may assist in positioning the substrate and facilitating the reaction. Interacting residues are shown in black (side-chain interactions) or blue (backbone interactions). The hydrophobic residues shown in gray line the entrance to the substrate pocket but do not interact directly with the bound angucyclinone. Note that one face of the pocket is formed by the cofactor itself (not shown). In the natural SimC7 substrate R=H, in the substrate used here R includes the deoxysugar, tetraene linker, and the aminocoumarin. (B) Orthogonal cross-sections through the active-site pocket, revealing how tightly cofactor (green) and substrate (yellow) are bound. For clarity, only the nicotinamide ribosyl moiety of the cofactor and the polyketide moiety of the substrate are shown. In (I) and (III) the view corresponds roughly to that shown in Figure 2D, whereas (II) and (IV) show the view from above relative to Figure 2D. In the lower panels, C-4 of the cofactor and C-7 of the substrate are highlighted by black spheres, showing that C-7 of the substrate is exactly positioned 3 Å from C-4 of the nicotinamide ring, poised for hydride transfer. See also Figures S1–S3.

Overall the apo, binary, and ternary SimC7 structures are very similar (Table S3), with the notable exception of the lid motif, which displays a number of different conformations (Figure 2C). Although the changes are not large (maximum Cα-Cα shift 5.35 Å; Table S3), there is a clear closure of the lid over the bound substrate (Figure 2C), suggesting a role in gating access to the active site and/or substrate capture. Moreover, in the ternary complex the underside of the lid contributes to the tight, highly hydrophobic substrate binding pocket (Figure 3) that provides the necessary environment for catalysis (see below).

### Structural Homologs of SimC7

Structures annotated as SDR proteins (PFAM family PF00106) are prevalent in the PDB, with more than 600 entries. To look for structural homologs of SimC7, we carried out a structure-based similarity search using the DALI server (Table S4). Strikingly, characterized angucyclinone ketoreductases ranked very low in the search, the closest match being LanV (Figure S2A), which was the 166^th^ ranked hit after filtering for sequence redundancy (Table S4). Instead, the two most structurally similar proteins to SimC7 were quinone oxidoreductase (QOR2) from *Escherichia coli* (PDB: 2ZCV) (Kim et al., 2008a) (Figure S2A) and triphenylmethane reductase (TMR) from *Citrobacter* sp. KCTC 18061P (PDB: 2VRB) (Kim et al., 2008b). QOR2 and TMR share with SimC7 the ability to reduce substrates with extensively conjugated pi systems but have roles in the detoxification of xenobiotics rather than the biosynthesis of natural products. The majority of the closest structural homologs of known function are involved in sugar biosynthesis, many of them epimerases.

SimC7 and its closest structural homologs all fall into the extended SDR subfamily of proteins, characterized by having two distinct domains that together form a partially occluded active-site pocket at their junction (Figure S2A). In contrast, the other structurally characterized polyketide ketoreductases such as LanV are more distantly related and belong to the classical SDR subfamily (Kavanagh et al., 2008), in which three insertions within the core Rossmann fold motif delineate a more accessible active-site cavity but do not constitute a well-defined substrate binding domain (Figure S2A).

### Substrate Binding

In the ternary complex with substrate, determined at 1.2-Å resolution, the angucyclic ring system of 7-oxo-SD8 binds adjacent and parallel to the nicotinamide ring of the cofactor (Figures 2D, 2E, and S3), where it adopts a relatively planar conformation differing from the conformations seen in the DNA gyrase-SD8 and SimR-SD8 complexes, in which the A ring of the angucyclinone in SD8 is tilted upward toward the epoxide (Hearnshaw et al., 2014, Le et al., 2011) (Figure S2B). This planar conformation is most likely enforced by the shape of the very constricted and highly hydrophobic substrate pocket (Figure 3). Within this hydrophobic pocket, 7-oxo-SD8 is bound only by a single direct hydrogen bond between the side chain of Ser95 and the C-7 carbonyl oxygen of the angucyclinone moiety, which may help to position the latter exactly above the C-4 position of the nicotinamide ring, where it is poised for direct hydride transfer (highlighted by black spheres in Figures 2C–2E, 3B, and S3). As mentioned above, the natural substrate for SimC7 is likely to be a 7-oxo angucyclinone intermediate lacking the deoxysugar, the tetraene linker, and the aminocoumarin. Consistent with this, only the angucyclinone moiety is buried in the active site of SimC7. Roughly half of the tetraene linker is visible in the electron density, projecting away from the protein surface, and the aminocoumarin ring is not resolved at all (Figure S3).

### A Novel Catalytic Mechanism for a Polyketide Ketoreductase

**Figure 4:**
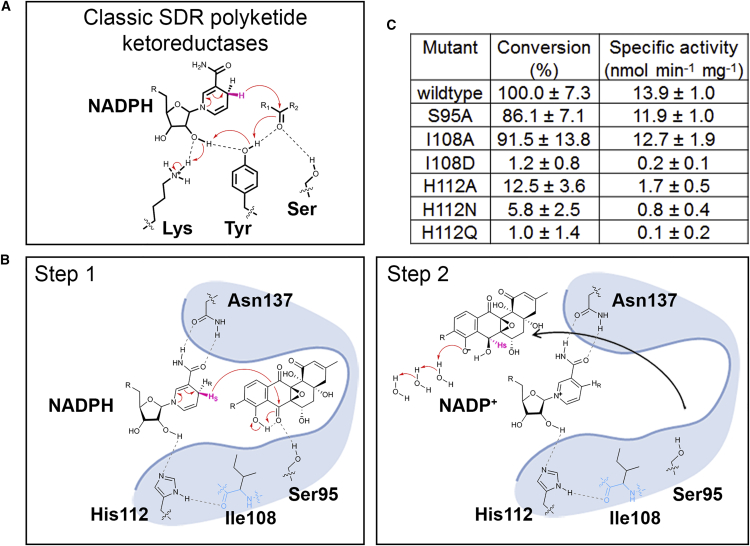
Canonical SDR Ketoreduction and the Novel SimC7 Reaction Mechanism (A) In canonical SDR proteins the conserved active-site tyrosine serves as central acid-base catalyst that donates a proton to the substrate. The adjacent lysine residue lowers the pK_a_ of the tyrosine hydroxyl group and often contributes directly to the proton relay mechanism; the hydroxyl group of the serine stabilizes and polarizes the carbonyl group of the substrate. (B) SimC7 has an atypical catalytic triad comprised of Ser95, Ile108, and His112. In the first step of the proposed SimC7 mechanism, the C-7 carbonyl group of the substrate (7-oxo-SD8) is reduced by transfer of the 4-pro-*S* hydride of the cofactor onto the C-7 carbon of the substrate. This transfer from below the C ring results in the characteristic 7-*S* stereochemistry of SD8. Ketoreduction at position C-7 is completed by an intramolecular proton transfer from the neighboring C-8 hydroxyl group of the angucyclinone; the resultant negative charge on the latter is stabilized by the adjacent aromatic ring system (ring D in Figure 1). In the second step, the C-8 phenolate intermediate regains a proton from bulk water after leaving the substrate binding pocket. In the natural SimC7 substrate R=H, in the substrate used here R includes the deoxysugar, tetraene linker, and the aminocoumarin. Note that there are no water molecules in the active-site pocket that could contribute to the reaction mechanism. In the ternary complex, the nearest water to O-7 of the angucyclic polyketide is ∼5.5 Å away, and the nearest water to O-8 is ∼4.9 Å away. Due to steric constraints within the pocket, neither could approach the substrate oxygen atoms without either a repositioning of the substrate or a conformational change in the protein. (C) Enzymatic activities of SimC7 active-site mutants. Standard errors are indicated for three independent experiments. See also Figures S3 and S4; Table S4.

SimC7 lacks the Ser-Tyr-Lys catalytic triad characteristic of canonical SDR proteins (Figure 4 and Table S4). While the serine is conserved (Ser95), the other two residues (i.e., the YxxxK motif) are not, being instead replaced by Ile108 and His112, respectively. This Ser-Ile-His triad is unlike any described for the five subfamilies of SDRs defined by Kavanagh et al. (2008). Particularly surprising is the absence of the tyrosine residue that acts as the acid-base catalyst in the classical SDR mechanism (Figure 4A). Inspection of the structure of the ternary complex shows that none of the five tyrosine residues in SimC7 is sufficiently close to C-7 of the angucyclinone ring system of the substrate to play a direct role in catalysis. Furthermore, the structure also shows that there is no alternative residue that could act as an acid-base catalyst. Consequently, SimC7 must perform ketoreduction of 7-oxo-SD8 via a novel mechanism.

Based on the structure of the ternary complex of the enzyme with NADP^+^ and 7-oxo-SD8, we propose a simple two-step mechanism for SimC7 that does not depend on catalytic residues in the protein, but rather takes advantage of the specific properties of the substrate itself, and is thus a novel example of substrate-assisted catalysis (Dall'Acqua and Carter, 2000). In the first step, the hydrophobic environment of the substrate binding pocket and the juxtaposition of the quinone-like C ring and the phenyl-like D ring of the angucyclinone favor the formation of an intramolecular hydrogen bond between the proton on the C-8 hydroxyl group and the oxygen of the neighboring C-7 carbonyl group (Figure 4B). This enhances the polarization of the latter such that the electrophilicity of C-7 is increased, making it a good acceptor for direct hydride transfer from the 4-*pro-S* position of the nicotinamide ring. Crucially, the hydride donor and acceptor carbon atoms are only 3.0 Å apart in the crystal structure. The C-7 hydroxyl group is then formed by internal proton transfer from the neighboring C-8 hydroxyl group, generating a phenolate intermediate in which the negative charge on the C-8 oxygen atom is stabilized by the aromatic D ring. In the second step of the reaction, the phenolate intermediate leaves the substrate binding pocket and the proton required to reinstate the C-8 hydroxyl group is recovered by abstraction from bulk water, which is not possible within the confines of the active site (Figure 4B). Exchange of the negatively charged reaction intermediate is most likely accelerated by repulsion from the hydrophobic active-site cavity. Finally, the direct hydride attack from below the angucyclic polyketide unambiguously explains the 7*S* stereochemistry of simocyclinones. In support of this proposed mechanism, molecular modeling predicts the existence of the key intramolecular hydrogen bond between the C-8 hydroxyl group and the C-7 carbonyl group of 7-oxo-SD8, and an equivalent intramolecular hydrogen bond is observed in the small-molecule crystal structure of panglimycin, a closely related polyketide (Fotso et al., 2008). Furthermore, molecular modeling also predicts that the C-8 hydroxyl group will have the most acidic and exchangeable proton in the angucyclic polyketide, with an estimated pK_a_ of 6.9–7.7, and therefore could readily transfer to the neighboring C-7 oxygen at the end of step 1. Indeed, there is no other possible proton donor (neither protein nor water derived) sufficiently close to O-7 to perform this role.

Based on their structures (Figure S4A), there are four other angucyclinones in which a SimC7-like mechanism might generate a C-7 hydroxyl group: panglimycin, elmycin, grisemycin, and kiamycin (Fotso et al., 2008, Xie et al., 2012, Xie et al., 2016). However, the biosynthetic gene clusters for these molecules have yet to be reported, and it is not known whether a SimC7-like enzyme is involved in their synthesis. Most other angucyclin(on)es have a carbonyl group at C-7.

### Mutagenesis of the SimC7 Active Site

To investigate the potential roles of the SimC7 “catalytic triad” residues in the proposed reaction mechanism, we mutagenized Ser95, Ile108, and His112 (Figure 4B). In the wild-type enzyme, the hydroxyl group of Ser95 could aid catalysis by helping to bind and correctly orient the substrate, and by providing additional polarization to the C-7 carbonyl group via a hydrogen bond, the latter role being consistent with the function proposed for the structurally equivalent Ser/Thr residues in the classical SDR mechanism (Figure 4A) (Kavanagh et al., 2008, Kallberg et al., 2010, Persson and Kallberg, 2013). However, neither effect would appear to be crucial for activity, as a S95A mutant showed 86% substrate conversion relative to the wild-type (Figure 4C). Therefore, SimC7 must be able to orient 7-oxo-SD8 for catalysis without the necessity for the hydrogen bond from Ser95, and this likely arises because the active-site cavity of the binary complex closely matches the shape of the substrate (Figure 3B).

Ile108 contributes to the hydrophobic surface that forms one side of the active-site cavity and has a nonspecific role in helping to trap the angucyclinone group against the nicotinamide ring of the cofactor (Figure 3B). Unsurprisingly, an I108A substitution had almost no effect, whereas changing it to aspartate (I108D) abolished activity (Figure 4C). In the latter case, the introduction of a negative charge would interfere with the hydrophobic environment and could disrupt the intramolecular hydrogen bond in the substrate, both being necessary for catalysis.

Finally, mutation of His112 (H112A, H112N, and H112Q) strongly reduced or abolished enzyme activity (Figure 4C), consistent with its key role in binding and positioning the cofactor via a hydrogen bond to the 2′-hydroxyl of the nicotinamide ribosyl moiety (Figures 2D, 2E, and 4B).

## Significance

**The sequence of SimC7 is distinct from previously characterized polyketide ketoreductases, and the structural data reported here suggest that it catalyzes a novel substrate-assisted, two-step reaction for the reduction of the C-7 carbonyl group. This mechanism involves the intramolecular transfer of a substrate-derived proton to generate a phenolate intermediate, negating the need for proton transfer from a canonical SDR active-site tyrosine. Like SimC7 (Ser-Ile-His), the two closest structural homologs, TMR (Tyr-Leu-His) and QOR2 (Leu-Leu-His), also have unusual active-site triads. Thus SimC7, TMR, and QOR2 share an I/LxxxH motif and have substrates with extensively conjugated pi systems. No enzyme-substrate complex crystal structures have been described for TMR or QOR2 and no firm proposals exist for their catalytic mechanisms, but their substrate structures and the data provided here suggest they are also likely to employ noncanonical mechanisms. Our data therefore point to members of the extended SDR sub-family having I/LxxxH active site motifs as a source of new biochemistry.**

## Experimental Procedures

For a full explanation of the experimental protocols, see Supplemental Experimental Procedures.

### Protein Overexpression and Purification

Point mutants of *simC7* were generated by PCR-based site-directed mutagenesis. All constructs were verified by sequencing. Proteins were expressed in *E. coli* as N-terminally His-tagged fusions, purified by nickel-affinity chromatography and assayed by high-performance liquid chromatography as described previously (Schäfer et al., 2015). The structural integrity of purified proteins was verified using circular dichroism.

### Protein Crystallization and Structure Determination

SimC7 was labeled with selenomethionine (SeMet) by metabolic inhibition, and crystals of both native and SeMet proteins were grown by vapor diffusion (crystals of binary and ternary complexes were obtained by cocrystallization). Crystals were harvested and flash-cooled in liquid nitrogen. The native crystals did not require further cryoprotection, while the SeMet-labeled crystals were cryoprotected by supplementing the crystallization solution with 25% (v/v) glycerol. All X-ray data were collected at the Diamond Light Source. For de novo structure determination, a single-wavelength anomalous dispersion dataset was collected at the Se *K* X-ray absorption edge for an SeMet-labeled SimC7 crystal. Native and SeMet data were combined to solve the structure of SimC7 in complex with NADP^+^. All other structures were determined by molecular replacement using the latter as a template.

## Author Contributions

All authors designed the experiments. M.S. and C.E.M.S. performed the experiments. All authors analyzed the data. M.S., D.M.L., and M.J.B. wrote the paper, and all authors made revisions.

## Accession Numbers

The accession numbers for the SimC7 structures reported in this paper are PDB: 5L40 (apo form 1), 5L45 (apo form 2), 5L3Z (binary complex), and 5L4L (ternary complex).

## Supplemental Information

Document S1. Supplemental Experimental Procedures, Figures S1–S4, and Tables S1–S4

Document S2. Article plus Supplemental Information

## References

[bib1] Collin F., Karkare S., Maxwell A. (2011). Exploiting bacterial DNA gyrase as a drug target: current state and perspectives. Appl. Microbiol. Biotechnol..

[bib2] Dall'Acqua W., Carter P. (2000). Substrate-assisted catalysis: molecular basis and biological significance. Protein Sci..

[bib3] Edwards M.J., Flatman R.H., Mitchenall L.A., Stevenson C.E.M., Le T.B.K., Clarke T.A., McKay A.R., Fiedler H.-P., Buttner M.J., Lawson D.M., Maxwell A. (2009). A crystal structure of the bifunctional antibiotic, simocyclinone D8, bound to DNA gyrase. Science.

[bib4] Edwards M.J., Williams M.A., Maxwell A., McKay A.R. (2011). Mass spectrometry reveals that the antibiotic simocyclinone D8 binds to DNA gyrase in a “bent-over” conformation: evidence of positive cooperativity in binding. Biochemistry.

[bib5] Fotso S., Mahmud T., Zabriskie T.M., Santosa D.A., Sulastri, Proteau P.J. (2008). Angucyclinones from an Indonesian *Streptomyces* sp. J. Nat. Prod..

[bib6] Hearnshaw S.J., Edwards M.J., Stevenson C.E., Lawson D.M., Maxwell A. (2014). A new crystal structure of the bifunctional antibiotic simocyclinone D8 bound to DNA gyrase gives fresh insight into the mechanism of inhibition. J. Mol. Biol..

[bib7] Javidpour P., Das A., Khosla C., Tsai S.C. (2011). Structural and biochemical studies of the hedamycin type II polyketide ketoreductase (HedKR): molecular basis of stereo- and regiospecificities. Biochemistry.

[bib8] Javidpour P., Korman T.P., Shakya G., Tsai S.C. (2011). Structural and biochemical analyses of regio- and stereospecificities observed in a type II polyketide ketoreductase. Biochemistry.

[bib9] Javidpour P., Bruegger J., Srithahan S., Korman T.P., Crump M.P., Crosby J., Burkart M.D., Tsai S.C. (2013). The determinants of activity and specificity in actinorhodin type II polyketide ketoreductase. Chem. Biol..

[bib10] Kallberg Y., Oppermann U., Persson B. (2010). Classification of the short-chain dehydrogenase/reductase superfamily using hidden Markov models. FEBS J..

[bib11] Kavanagh K.L., Jörnvall H., Persson B., Oppermann U. (2008). The SDR superfamily: functional and structural diversity within a family of metabolic and regulatory enzymes. Cell. Mol. Life Sci..

[bib12] Kharel M.K., Pahari P., Shepherd M.D., Tibrewal N., Nybo S.E., Shaaban K.A., Rohr J. (2012). Angucyclines: biosynthesis, mode-of-action, new natural products, and synthesis. Nat. Prod. Rep..

[bib13] Kim I.K., Yim H.S., Kim M.K., Kim D.W., Kim Y.M., Cha S.S., Kang S.O. (2008). Crystal structure of a new type of NADPH-dependent quinone oxidoreductase (QOR2) from *Escherichia coli*. J. Mol. Biol..

[bib14] Kim M.H., Kim Y., Park H.J., Lee J.S., Kwak S.N., Jung W.H., Lee S.G., Kim D., Lee Y.C., Oh T.K. (2008). Structural insight into bioremediation of triphenylmethane dyes by *Citrobacter* sp. triphenylmethane reductase. J. Biol. Chem..

[bib15] Korman T.P., Hill J.A., Vu T.N., Tsai S.C. (2004). Structural analysis of actinorhodin polyketide ketoreductase: cofactor binding and substrate specificity. Biochemistry.

[bib16] Korman T.P., Tan Y.H., Wong J., Luo R., Tsai S.C. (2008). Inhibition kinetics and emodin cocrystal structure of a type II polyketide ketoreductase. Biochemistry.

[bib17] Le T.B.K., Stevenson C.E.M., Fiedler H.-P., Maxwell A., Lawson D.M., Buttner M.J. (2011). Structures of the TetR-like simocyclinone efflux pump repressor, SimR, and the mechanism of ligand-mediated derepression. J. Mol. Biol..

[bib18] Paananen P., Patrikainen P., Kallio P., Mäntsälä P., Niemi J., Niiranen L., Metsä-Ketelä M. (2013). Structural and functional analysis of angucycline C-6 ketoreductase LanV involved in landomycin biosynthesis. Biochemistry.

[bib19] Patrikainen P., Niiranen L., Thapa K., Paananen P., Tähtinen P., Mäntsälä P., Niemi J., Metsä-Ketelä M. (2014). Structure-based engineering of angucyclinone 6-ketoreductases. Chem. Biol..

[bib20] Persson B., Kallberg Y. (2013). Classification and nomenclature of the superfamily of short-chain dehydrogenases/reductases (SDRs). Chem. Biol. Interact..

[bib21] Schäfer M., Le T.B.K., Hearnshaw S.J., Maxwell A., Challis G.L., Wilkinson B., Buttner M.J. (2015). SimC7 is a novel NAD(P)H-dependent ketoreductase essential for the antibiotic activity of the DNA gyrase inhibitor simocyclinone. J. Mol. Biol..

[bib22] Schimana J., Fiedler H.P., Groth I., Sussmuth R., Beil W., Walker M., Zeeck A. (2000). Simocyclinones, novel cytostatic angucyclinone antibiotics produced by *Streptomyces antibioticus* Tu 6040. I. Taxonomy, fermentation, isolation and biological activities. J. Antibiot. (Tokyo).

[bib23] Schimana J., Walker M., Zeeck A., Fiedler H.-P. (2001). Simocyclinones: diversity of metabolites is dependent on fermentation conditions. J. Ind. Microbiol. Biotechnol..

[bib24] Trefzer A., Pelzer S., Schimana J., Stockert S., Bihlmaier C., Fiedler H.-P., Welzel K., Vente A., Bechthold A. (2002). Biosynthetic gene cluster of simocyclinone, a natural multihybrid antibiotic. Antimicrob. Agents Chemother..

[bib25] Xie Z., Liu B., Wang H., Yang S., Zhang H., Wang Y., Ji N., Qin S., Laatsch H. (2012). Kiamycin, a unique cytotoxic angucyclinone derivative from a marine *Streptomyces* sp. Mar. Drugs.

[bib26] Xie Z., Zhou L., Guo L., Yang X., Qu G., Wu C., Zhang S. (2016). Grisemycin, a bridged angucyclinone with a methylsulfinyl moiety from a marine-derived *Streptomyces* sp. Org. Lett..

