# Supplementary material for: Substrate-Assisted Catalysis in Polyketide Reduction Proceeds via a Phenolate Intermediate
Source: Cell Chem Biol. 2016 Sep 22;23(9):1091–7. doi: 10.1016/j.chembiol.2016.07.018 (PMC5039031; doi:10.1016/j.chembiol.2016.07.018)
Supplement: Document S1. Supplemental Experimental Procedures, Figures S1–S4, and Tables S1–S4 [file mmc1.pdf]

Cell Chemical Biology, Volume *23*

Supplemental Information

Substrate-Assisted Catalysis in Polyketide Reduction Proceeds via a Phenolate Intermediate

Martin Schäfer, Clare E.M. Stevenson, Barrie Wilkinson, David M. Lawson, and Mark J. Buttner

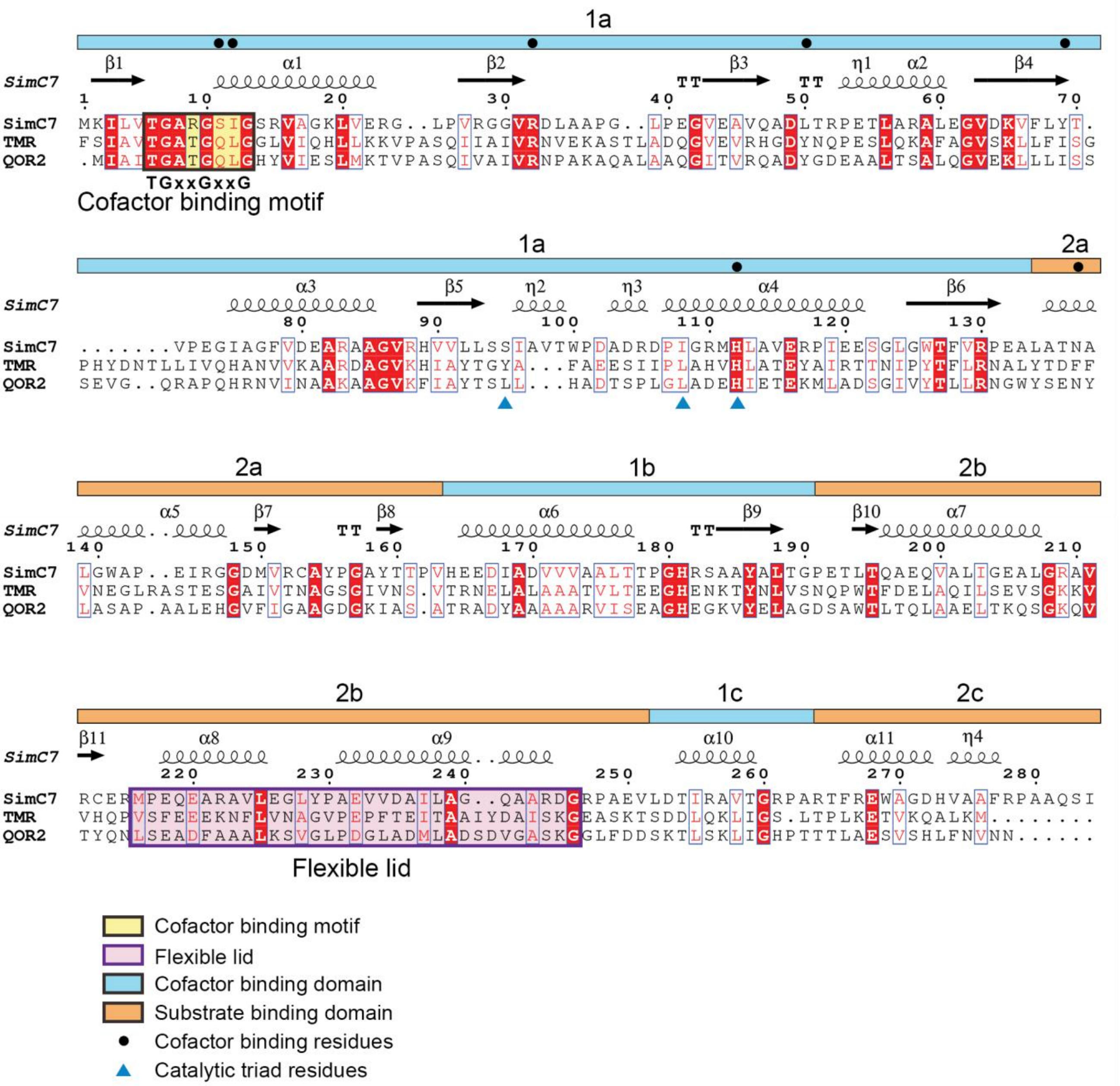

Figure S1, related to Figure 2. Structure-based sequence alignment of SimC7 with triphenylmethane reductase (TMR) from *Citrobacter* sp. and quinone oxidoreductase (QOR2) from *E. coli*. Conserved residues are shown in white on a red background and similar residues are shown in red font. Secondary structure for SimC7 is shown above the alignment (α for α-helix and β for β-strand, TT for β-turn and η for 3_{10} helix). The bars above the alignment indicate the cofactor binding domain (1a-c in blue) and the substrate binding domain (2a-c in orange) with black dots indicating residues that interact with the $NADP^+$ cofactor. The conserved N-terminal cofactor-binding motif (TGxxGxxG, yellow with black frame) and the flexible lid (Met216 to Gly246, pink frame) are highlighted. Blue triangles indicate the positions equivalent to the active site triad residues in canonical SDR proteins.

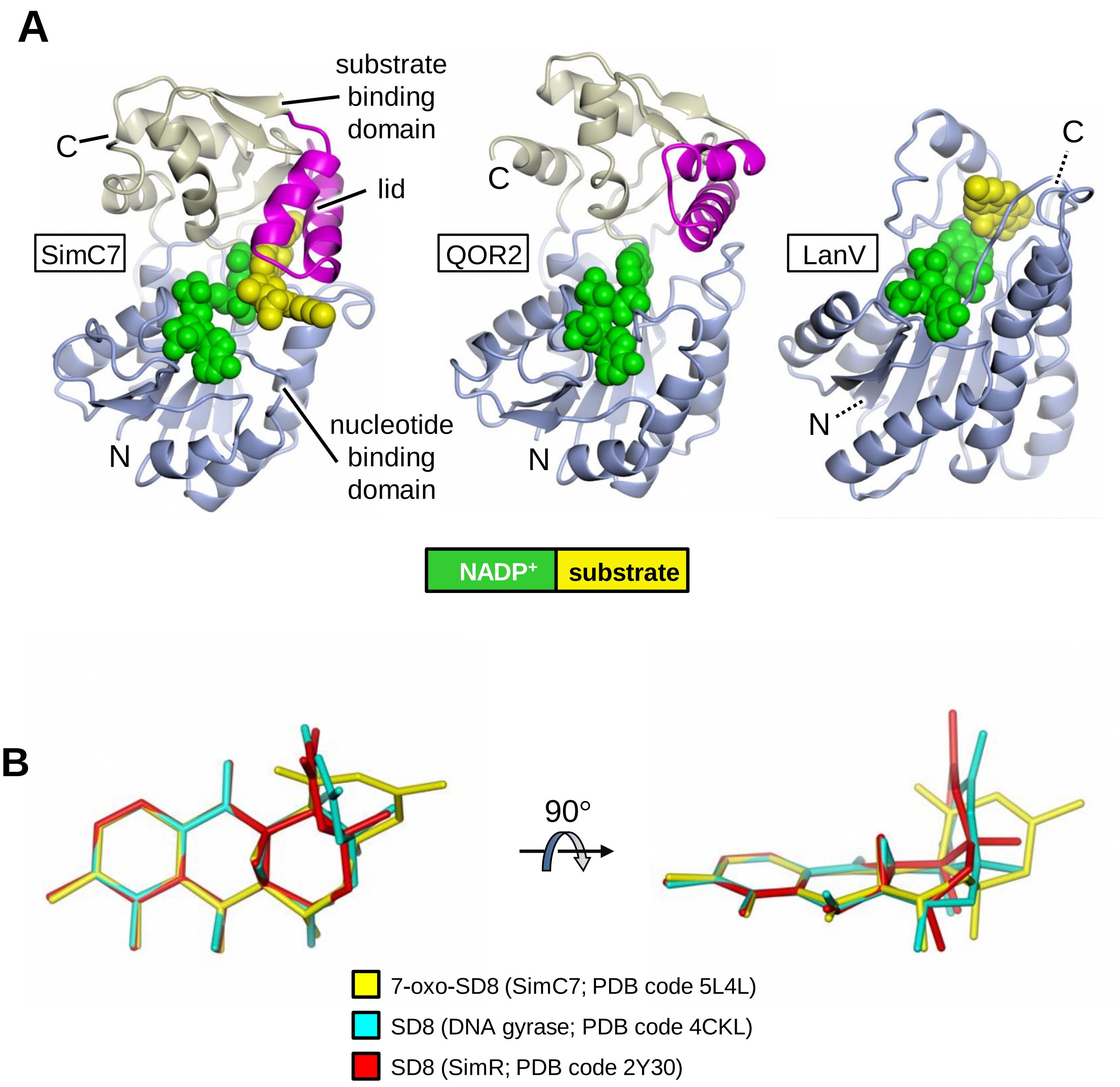

Figure S2, related to Figure 2. (A) Comparison of the ternary complexes of SimC7 and LanV, and the binary complex of QOR2. Structures are depicted in cartoon representation, where pale blue, beige and magenta colouration indicate the nucleotide binding domain, the substrate binding domain and the lid motif, respectively (N.B. there is no distinct substrate binding domain or lid motif in LanV). The ligands are shown as van der Waals spheres, with the $NADP^+$ cofactor shown in green and the substrate (where present) shown in yellow. See Table S4 for a more extensive comparison of SimC7 structural homologues. (B) Comparison of the 7-oxo-SD8 conformation in the ternary complex of SimC7 with SD8 conformations observed previously in other complexes. Orthogonal views showing a least-squares superposition of the angucyclic moiety of 7-oxo-SD8 (based on the 6 carbon atoms of ring D) upon SD8 from the complex with GyrA55 and from the complex with SimR.

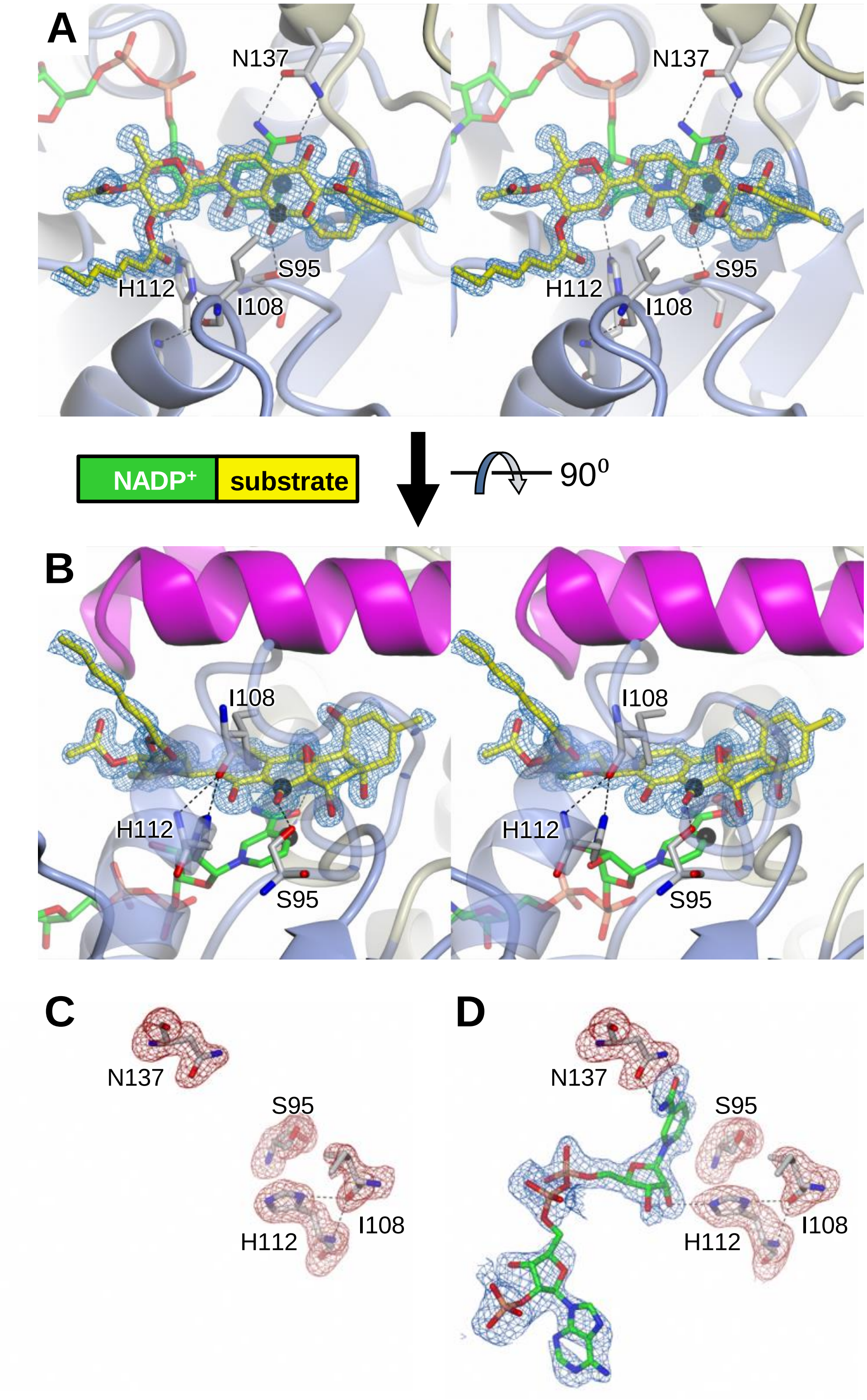
A
N137
N137
H112
I108
S95
H112
I108
S95
NADP+
substrate
90⁰
B
I108
I108
H112
S95
H112
S95
C
D
N137
S95
I108
H112
N137
S95
I108
H112

Figure S3, related to Figure 2. Simulated annealing omit electron density maps. Parts (A) and (B) show orthogonal stereo views of the active site of the SimC7 ternary complex. Ligands are depicted in stick representation, where green indicates carbon atoms of the cofactor and yellow, carbons of the substrate; C-4 of the cofactor and C-7 of the substrate are specifically highlighted by small black spheres. Superposed upon the substrate in blue is a simulated annealing omit map calculated at 1.2 Å resolution (see Extended Experimental Procedures). Also shown are the catalytic triad residues, as well as Asn137, which is important in maintaining the *syn* conformation of the cofactor. The view in part (A) is equivalent to that in Fig. 2E (again the lid motif has been removed for clarity). In part (B), the protein backbone encompassing the β5 - α4 loop and bearing the catalytic triad has been shown in transparent mode as it would otherwise obscure the detail of the active site; the lid is in magenta. Parts (C) and (D) show omit maps (red density) for just the active site residues in Apo form 1 (1.6 Å resolution) and the binary complex (1.95 Å resolution), respectively. In the latter panel a separate omit map is also shown (blue density), which was calculated from the final model lacking only the cofactor. The view in parts (C) and (D) is roughly equivalent to that used in Fig. 2D. All omit maps were contoured at ~3.0 σ.

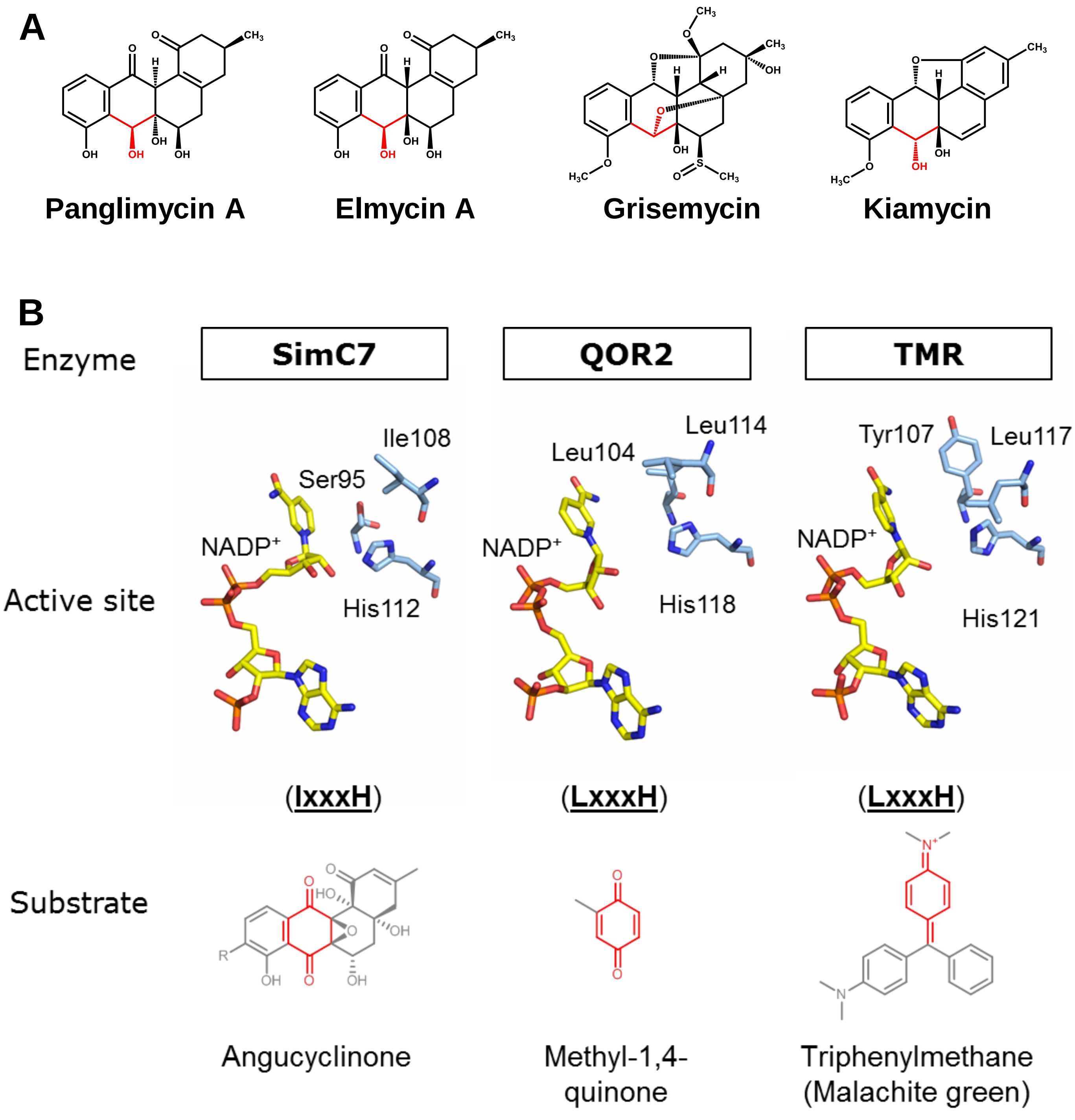

Figure S4, related to Figure 4. (A) Structures of angucyclinones in which a SimC7-like mechanism might generate a C-7 hydroxyl group. Panglimycin, elmycin, grisemycin and kiamycin are all produced by *Streptomyces* strains but their biosynthetic gene clusters have not been reported. The unusual intramolecular ether bridge between C-4a and C-7 in grisemycin is most likely formed from a precursor having hydroxyl groups at both positions. (B) Variations in the active site triad and substrates between SimC7, QOR2 and TMR. Arrangement of the cofactor (yellow) and the protein residues (blue) that constitute the active site triad in SimC7, quinone oxidoreductase (QOR2, PDB accession number 2ZCV) from *E. coli*, and triphenylmethane reductase (TMR, PDB accession number 2JL1) from *Citrobacter* sp. The substrate of each enzyme is shown below. The natural substrate of TMR is not known.

Table S1, related to Figure 2. X-ray data collection and processing

Data set	Binary SeMet	Binary native	Apo form 1	Apo form 2	$NADP^+$ plus 7-oxo ternary complex
Beamline	I03	I03	I04-1	I04-1	I04
Wavelength (Å)	0.9796	1.0052	0.9173	0.9173	0.9795
Detector	Pilatus 6M	Pilatus 6M	Pilatus 6M	Pilatus 6M	Pilatus 6M
Resolution range (Å)[a]	51.48 – 2.05 (2.10 – 2.05)	50.74 – 1.95 (2.00 – 1.95)	53.40 – 1.60 (1.64 – 1.60)	54.88 – 1.90 (1.95 – 1.90)	47.52 – 1.20 (1.23 – 1.20)
Space Group	$P4_12_12$	$P4_12_12$	$P4_12_12$	C2	$P2_12_12_1$
a, b, c (Å)	51.5, 51.5, 213.2	52.2, 52.2, 214.0	52.4, 52.4, 213.6	107.0, 64.8, 91.5	51.9, 53.7, 102.3
α, β, γ (°)	90.0, 90.0, 90.0	90.0, 90.0, 90.0	90.0, 90.0, 90.0	90.0, 105.5, 90.0	90.0, 90.0, 90.0
Total observations[a]	457751 (25343)	563166 (40131)	435288 (32092)	336053 (23051)	1159483 (81348)
Unique reflections[a]	19033 (1335)	22650 (1625)	40514 (2936)	47271 (3433)	90009 (6590)
Multiplicity[a]	24.1 (19.0)	24.9 (24.7)	10.7 (10.9)	7.1 (6.7)	12.9 (12.3)
Mean $I/\sigma(I)$[a]	16.5 (1.3)	18.4 (2.2)	20.0 (1.5)	13.6 (1.0)	17.5 (2.1)
Completeness (%)[a]	99.8 (99.7)	99.9 (99.8)	99.9 (99.2)	98.5 (96.1)	100.0 (99.5)
R_{merge}[a,b]	0.122 (2.745)	0.118 (1.845)	0.066 (1.814)	0.087 (1.990)	0.070 (1.208)
R_{meas}[a,c]	0.125 (2.820)	0.120 (1.883)	0.069 (1.903)	0.094 (2.157)	0.073 (1.249)
$CC_{½}$[a,d]	0.999 (0.673)	0.999 (0.819)	1.000 (0.701)	0.999 (0.543)	0.999 (0.760)
Wilson *B* value ($Å^2$)	45.3	34.0	21.6	34.2	11.9

[a] Values for the outer resolution shell are given in parentheses.
[b] $R_{merge} = \sum_{hkl} \sum_i |I_i(hkl) - \langle I(hkl)\rangle| / \sum_{hkl} \sum_i I_i(hkl)$.
[c] $R_{meas} = \sum_{hkl} [N/(N-1)]^{1/2} \times \sum_i |I_i(hkl) - \langle I(hkl)\rangle| / \sum_{hkl} \sum_i I_i(hkl)$, where $I_i(hkl)$ is the ith observation of reflection hkl, $\langle I(hkl)\rangle$ is the weighted average intensity for all observations i of reflection hkl and N is the number of observations of reflection hkl.
[d] $CC_{½}$ is the correlation coefficient between symmetry-related intensities taken from random halves of the dataset.

Table S2, related to Figure 2. Refinement of X-ray structures

Data set	Binary native	Apo form 1	Apo form 2	NADP[+] plus 7-oxo ternary complex
Resolution range (Å)[a]	50.74 – 1.95 (2.00 – 1.95)	53.40 – 1.60 (1.64 – 1.60)	54.88 – 1.90 (1.95 – 1.90)	47.52 – 1.20 (1.23 – 1.20)
Reflections: working/free[b]	21479/1171	38504/2010	44913/2357	85532/4476
Final R_{work}[a,c]	0.182 (0.282)	0.189 (0.297)	0.195 (0.342)	0.126 (0.218)
Final R_{free}[a,c]	0.217 (0.316)	0.218 (0.303)	0.229 (0.348)	0.149 (0.230)
Cruickshank DPI (Å)[d]	0.139	0.087	0.150	0.032
R.m.s. bond deviations (Å)	0.010	0.010	0.009	0.010
R.m.s. angle deviations (°)	1.40	1.38	1.29	1.52
No. of protein residues (ranges)	280 (1 to 280)	280 (1 to 280)	A chain:278 (2-279); B chain 281 (-1 to 279)[*]	278 (2-279)
No. of heterogen residues: cofactor/ 7-oxo/water/other	1/0/104/0	0/0/167/0	0/0/202/2	1/1/324/1
Mean *B*-factors: protein/cofactor/ 7-oxo/water/other/overall (Å^2)	47/46[#]/-/46/-/47	35/-/-/39/-/35	50/-/-/49/58/50	17/11/21/32/28/19
Ramachandran plot: favoured/allowed/ disallowed (%)[e]	98.6/1.4/0.0	99.3/0.7/0.0	98.4/1.2/0.4	99.1/0.9/0.0
PDB accession code	5L3Z	5L40	5L45	5L4L

[a] Values for the outer resolution shell are given in parentheses.
[b] The data set was split into "working" and "free" sets consisting of 95 and 5% of the data, respectively. The free set was not used for refinement.
[c] The R-factors R_{work} and R_{free} are calculated as follows: $R = \sum(|F_{obs} - F_{calc}|)/\sum|F_{obs}|$, where F_{obs} and F_{calc} are the observed and calculated structure factor amplitudes, respectively.
[d] Diffraction precision indicator based on R_{free} based as calculated by *REFMAC5* (Murshudov et al., 1997).
[e] As calculated using *MOLPROBITY* (Davis et al., 2007).
[*] Two residues were visible for the N-terminal His-tag in the B-chain. Since the numbering scheme was based on the wild-type sequence, these residues were labelled as “-1” and “0”.
[#] Cofactor was refined with occupancy of 0.7.

Table S3 related to Figure 2. Comparison of SimC7 structures

Overall r.m.s. deviations (Å) [maximum Cα shift (Å)/ corresponding residue]	Apo form 1	Apo form 2: A chain	Apo form 2: B chain	Binary complex	Ternary complex
Apo form 1	0.00	1.13 [4.18/P37]* [3.82/A231]	0.58 [1.71/P230]	0.18‡ [0.40/R222]	0.84 [3.67/G227]
Apo form 2: A chain		0.00	0.88 [3.15/E232]	1.10 [3.92/A231]	1.43 [5.35/G227]
Apo form 2: B chain			0.00	0.58 [1.65/P230]	0.98 [4.20/G227]
Binary complex				0.00	0.83 [3.67/G227]
Ternary complex					0.00

Pairwise superpositions of all protein structures determined herein; R.m.s. deviations were determined by LSQKAB [Kabsch, 1976]. For Apo form 2, the two molecules in the ASU were treated as separate models.

*With the exception of this comparison, all the others showed the biggest shift in the lid region (residues 216-246 inclusive). Here the biggest shift was due to a *trans/cis* isomerisation of Pro37. The second largest shift (in the lid) is also shown.

‡ N.B. Apo form 1 and the binary complex are isomorphous.

Table S4, related to Figure 2. Selected structural homologues of SimC7

Protein	Source	Biological unit[a]	PDB code[b]	Ligand bound[c]	Resolution (Å)	DALI output					"Catalytic" triad	Reference
						Rank[d]	Z-score	R.m.s.d. (Å)	aligned residues	Identity (%)		
SimC7	*Streptomyces antibioticus* Tü6040	Monomer	5L4L	NADP± & 7oxo-SD8	1.20	-	-	0.00	278	100	Ser95, Ile108, His112	This work
QOR2	*Escherichia coli*	Monomer	2ZCV	$NADP^+$	1.60	1	27.4	3.1	266	23	Leu104, Leu114, His118	Kim *et al*., 2008
TMR	*Citrobacter* sp. KCTC 18061P	Dimer	2JL1	$NADP^+$	1.96	2	27.0	3.2	268	22	Tyr107, Leu117, His121	Kim *et al*., 2008
HSCARG	*Homo sapiens*	Monomer/ Dimer	2EXX	NADP±	2.40	4	24.8	3.1	263	22	Leu114, His129, Lys133	Zheng *et al*., 2007
UDP-hexose 4-epimerase	*Thermotoga maritima*	Dimer	4ZRM	NADP± & UDP-Glc	2.00	6	22.9	2.5	247	20	Thr117, Tyr143, Lys147	Shin *et al*., 2015
Cinnamoyl-CoA reductase	*Petunia hybrid*	(Monomer)	4R1S	NADP±	1.60	8	22.4	3.0	246	22	Ser123, Tyr157, Lys161	Pan *et al*., 2014
LanV	*Streptomyces cyanogenus*	Dimer	4KWI	NADP± & Lando	2.00	166	14.3	3.0	173	22	Ser147, Tyr160, Lys164	Paananen *et al*., 2013
UrdMred	*Streptomyces fradiae*	(Tetramer)	4OSP	NADP± & rabelomycin	2.25	167	14.3	3.2	177	20	Ser147, Tyr160, Lys164	Patrikainen *et al*., 2104
HedKR	*Streptomyces griseoruber*	(Tetramer)	3SJU	NADP±	2.40	N/A	15.5	4.0	185	19	Ser142, Tyr155, Lys159	Javidpour *et al*., 2011
ActKR	*Streptomyces coelicolor*	Tetramer	2RHC	NADP± & Emodin	2.10	N/A	15.1	3.9	185	18	Ser144, Tyr157, Lys161	Korman *et al*., 2008

[a] Experimentally determined (e.g. by size exclusion chromatography), unless shown in brackets, in which case it was inferred from the crystal structure alone. For HSCARG, the oligomeric state (as judged by dynamic light scattering) was dependent on the $NADP^+$ concentration.
[b] Results of a DALI search (http://ekhidna.biocenter.helsinki.fi/dali_server) (Holm and Sander, 1995) using the SimC7 ternary complex structure as the template. The hits were filtered for redundancy; where a relevant ligand bound structure exists for a particular enzyme, this is the one that is shown.
[c] Lando = 11-deoxy-6-oxylandomycinone. Where the cofactor is underlined, this indicates that the nicotinamde ring adopts the *syn* configuration like SimC7; otherwise the configuration is *anti*.
[d] Ranking of the DALI hit (after redundancy filtering at 90% sequence identity). For some classes of enzymes, e.g. the sugar epimerases, only the top hit is shown. The lower ranking hits LanV and UrdMred were also chosen as they were relevant to the discussion. For the same reason, ActKR and HedKR were included, despite not being picked up by a blind DALI search.

SUPPLEMENTAL EXPERIMENTAL PROCEDURES

Site-directed mutagenesis of *simC7*

The PCR-based Q5® site-directed mutagenesis kit [New England Biolabs] was used to generate *simC7* point mutants, using plasmid pET15b-NB-C7 as the template. Pairs of oligonucleotides were designed to amplify linearized plasmid DNA with one oligonucleotide containing the desired mutation. The template plasmid was degraded with *Dpn*I and the linear PCR product was self-ligated in the presence of kinase and DNA ligase before transformation into *E. coli* DH5α. Mutations were verified by DNA sequencing.

Oligonucleotides used. Mutant sites are underlined and in uppercase.

Oligonucleotide	Mutation	Sequence (5'-3')
S95A-F	S95A	tgctctccGCCatcgccgtgacctggc
S95AT-R	S95A	gcaccacatgccggaccc
I108A-F	I108A	gacccgGCGggccggatgcacctc
I108D-F	I108D	gacccgGATggccggatgcacctc
I108A-R	I108A/D	ccggtccgcgtccggccaggtc
H112A-F	H112A	ggatgGCCctcgccgtcga
H112N-F	H112N	ggatgAACctcgccgtcgagcg
H112Q-F	H112Q	ggatgCAGctcgccgtcgagcg
H112R-R	H112A/N/Q	ggccgatcgggtcccgg

Protein overexpression and purification

N-terminally His_6-tagged SimC7 and its mutant derivatives were expressed from pET15b-NB-C7 in *E. coli* Rosetta(DE3) pLysS cells (Schäfer *et al.*, 2015). The recombinant protein had 20 additional amino acids at the N-terminus compared to the native sequence (MGSSHHHHHHSSGLVPRGSH) and a molecular weight of 32,235 Da. We established from a number of expression and purification trials that the presence of sodium chloride had a tendency to induce aggregation of protein derived from this construct. For this reason, sodium chloride was either excluded or kept at low concentrations in the buffers used thereafter. 10 mL overnight culture was used to inoculate 1 L of Luria-Bertani medium without sodium chloride containing 40 mg carbenicillin and 10 mg chloramphenicol. The culture was grown at 37 °C to OD_{600} ~0.3, cooled to 18 °C, and protein expression was induced by addition of IPTG to a final concentration of 0.5 mM. The culture was incubated for 16 h shaking at 18 °C. Harvested cells were resuspended in lysis buffer [20 mM Na-HEPES (pH 8.0), 40 mM NaCl, 10% (v/v) glycerol, 10 mM imidazole] containing complete EDTA-free protease inhibitor cocktail (Roche), and lysed in a cell disruptor (three cycles with 16,000 psi pressure). Cell debris was removed by centrifugation at 40,000 *g* for 30 min, the supernatant was mixed with 1 mL of Ni-NTP-Agarose beads (Qiagen), and the resin was packed into a 5-mL Polypropylene column (Qiagen) equilibrated with wash buffer [20 mM Na-HEPES (pH 8.0), 40 mM NaCl, 10% (v/v) glycerol, 10 mM imidazole]. The column was washed with 700 mL wash buffer and SimC7 was eluted in 10 mL elution buffer [20 mM Na-HEPES (pH 8.0), 40 mM NaCl, 20% (v/v) glycerol, and 300 mM imidazole]. Protein samples were analysed by SDS-PAGE and immediately exchanged into storage buffer [20 mM Na-HEPES (pH 8.0), 10% (v/v) glycerol, 5 mM (w/v) TCEP] and concentrated to 4 mg/mL using a Vivaspin 15 buffer exchange column (Satorius Stedim Biotech, Germany). The CD spectra of all the mutant SimC7 proteins were collected (see below) and found to be unchanged relative to that of the wild-type enzyme.

For *de novo* structure determination, SimC7 was labelled with selenomethionine (SeMet) by metabolic inhibition (Doublie, 1997). Cells were grown in 2x 0.5 L M9 minimal medium at 37°C to OD_{600} ~0.1, amino acids were added (100mg/L each of threonine, lysine and phenylalanine; 50 mg/L each of leucine, isoleucine and valine; 60 mg/L SeMet), and the cultures were further incubated for 90 min. Protein overexpression was induced with 0.5 mM IPTG overnight at 18°C. Labelled protein was purified as described above.

Circular Dichroism

Circular dichroism (CD) spectroscopy experiments were performed using a Chirascan-Plus CD spectrophotometer (Applied Photophysics, Surrey, UK). Concentrated proteins in storage buffer were buffer exchanged against 20 mM potassium phosphate buffer (pH 7.2) with 5% (v/v) glycerol and diluted to 0.2 mg/mL (6.25 µM). CD analysis was carried out at 20 °C using a quartz glass cuvette with a 0.5 mm path length. CD spectra were averaged from four scans collected between 190 nm and 260 nm wavelength, using a bandwidth of 2.0 nm, a step size of 0.5 nm, and one time point per second.

SimC7 ketoreductase assays

SimC7 variants were assayed by UV/vis-HPLC using 500 nM SimC7, 0.3 mM NADH and 0.2 mM 7-oxo-SD8, as described previously (Schäfer *et al*., 2015). Purified SD8 and 7-oxo-SD8 were used as standards. Substrate conversion was determined by quantification of the peak area for the reaction product SD8 based on a serial dilution of SD8 prepared in methanol (0.25-300 µM). Samples (30 µL) were separated on a reverse-phase HPLC column (Phenomenex Gemini-NX 3u C18 110A, 150 mm × 4.6 mm) using a linear gradient of 70-95% (v/v) methanol against 0.1% (v/v) formic acid over 10 min, followed by 95% (v/v) methanol for 5 min at a flow rate of 1 mL/min. Assays were performed in triplicate using the average of two technical replicates for each data point.

Protein crystallization and cryoprotection

All crystallization experiments were performed with N-terminally His-tagged SimC7 at a concentration of approximately 4 mg/mL and at a temperature of 20°C. Screening was conducted by sitting-drop vapor diffusion in MRC 96-well crystallization plates (Molecular Dimensions) with a mixture of 0.3 µl well solution (from both commercial and in-house screens) and 0.3 µl protein solution using an OryxNano crystallization robot (Douglas Instruments). Promising conditions were optimised manually in 24-well hanging-drop vapor diffusion format using XRL plates (Molecular Dimensions) with drops consisting of 1 µl protein and 1 µl precipitant. Tetragonal crystals of the binary complex with $NADP^+$ were obtained from 16% (w/v) PEG-8000 and 20% (v/v) glycerol (native SimC7) or 1.3 M DL-malic acid (pH 7) supplemented with a final concentration of 1% (v/v) DMSO and 2.5 mM EDTA in the drop (SeMet-labelled SimC7). The native crystals did not require further cryoprotection, while the SeMet crystals were cryoprotected by supplementing the crystallization solution with 25% (v/v) glycerol. Crystals of *apo*-SimC7 that were isomorphous with those of the binary complex (apo form 1) grew from 25% (w/v) PEG-6000, 20% (v/v) glycerol in 0.1 M MES pH 6.5, while monoclinic *apo*-SimC7 crystals (apo form 2) were obtained from 16% (w/v) PEG-8000, 18% (v/v) glycerol with 10 mM dithiothreitol (DTT) added to the drop; neither form required further cryoprotection. 7-oxo-SD8 was isolated from *S. antibioticus* Tü6040 as described previously (Schäfer et al., 2015), and was dissolved in DMSO to a concentration of 50 mM prior to mixing with protein. The cofactor $NADP^+$ (Sigma) was dissolved in water to a concentration of 100 mM without adjusting the pH, which was crucial for obtaining crystals. SimC7 was first mixed with $NADP^+$ and then with 7-oxo-SD8, giving a final concentration of 0.12 mM (3.8 mg/mL) protein, 1.5 mM of 7-oxo-SD8, and 3.5 mM of $NADP^+$. The final concentration of DMSO did not exceed 3% (v/v). Orthorhombic crystals of the ternary complex of SimC7 bound to $NADP^+$ and 7-oxo-SD8 grew from 20-25% (w/v) PEG-8000, 20-25% (v/v) glycerol in 0.1 M sodium acetate pH 4.8, and did not require further cryoprotection.

X-ray data collection, structure determination and refinement

Crystals were harvested and flash-cooled in liquid nitrogen using LithoLoops (Molecular Dimensions). The mounted crystals were stored in Unipuck cassettes (MiTeGen) prior to transport to the Diamond Light Source (Oxfordshire, UK), where they were transferred robotically to the goniostat on either beamline I03, I04 or I04-1 and maintained at -173 °C with a Cryojet cryocooler (Oxford Instruments). X-ray diffraction data were recorded using a Pilatus 6M hybrid photon counting detector (Dectris), then integrated using XDS (Kabsch, 2010), and scaled and merged using AIMLESS (Evans and Murshudov, 2013) via the XIA2 expert system (Winter, 2010); the resultant data collection statistics are summarized in Table S1.

Native data from the binary complex with $NADP^+$ were collected to 1.95 Å and processed in space group $P4_12_12$ with an estimated solvent content of 49.3%, based on one copy of the protein chain in the ASU. A SeMet-labelled SimC7 crystal was used to collect a single-wavelength anomalous dispersion data set at the selenium *K* X-ray absorption edge (wavelength 0.9796 Å). The data were processed to 2.05 Å resolution in space group $P4_12_12$ and were isomorphous with the native set. Experimental phases were determined by analyzing the SeMet and native data using the SHELX suite (Sheldrick, 2008). SHELXD located 4 selenium sites and, after phasing with SHELXE (which revealed that $P4_12_12$ was the correct enantiomorph), the figure-of-merit was 0.589 to 1.95 Å resolution. These phases were improved by density modification with PARROT (Cowtan, 2010) and used as input to automated building with BUCCANEER (Cowtan, 2006), which was able to fit 233 residues (82% of the native sequence). After combining phases calculated from this model with the original experimental phases using SIGMAA (Read, 1986), PARROT and BUCCANEER were re-run. This time 277 residues (98% of the native sequence) were fitted giving R_{work} and R_{free} values of 0.263 and 0.298, respectively, at 1.95 Å resolution. The model of the binary complex was completed by several iterations of manual rebuilding in COOT (Emsley and Cowtan, 2004) and restrained refinement in REFMAC5 (Murshudov et al., 1997) using isotropic thermal parameters and TLS group definitions obtained from the TLSMD server (Painter and Merritt, 2006). The statistics of the final model are reported in Table S2.

A dataset was collected to 1.60 Å resolution from apo-SimC7 crystallized in the same form as the binary complex. This was used to generate the model of apo form 1 starting from the model of the binary complex from which the coordinates of the cofactor had been removed. Model building and refinement were completed as above. Crystals of apo form 2 yielded a dataset to 1.90 Å resolution after processing in space group C2, with an estimated solvent content of 48.3% based on two copies of the protein in the ASU. The structure was solved by molecular replacement with PHASER

(McCoy et al., 2007) using the protein component of binary complex structure as the template, and then refined as above.

Data for the ternary complex of SimC7 with $NADP^+$ and 7-oxo-SD8 were processed to 1.20 Å resolution in space group $P2_12_12_1$, giving an estimated solvent content of 45.3% based on one copy of the protein in the ASU. The latter was located using PHASER with the same template as before. For this structure it was possible to use anisotropic thermal parameter refinement in REFMAC5.

Model geometries were validated with MOLPROBITY (Davis et al., 2007) before submission to the Protein Data Bank. The statistics of all final models are summarized in Table S2.

A simulated annealing omit procedure was used to give an unbiased representation of the electron density for specific parts of the structures. For these, the part in question (e.g. the substrate in the ternary complex) was deleted from the coordinates of the final model. The resultant PDB file was used as input to simulated annealing refinement with PHENIX (Adams et al., 2010) from a starting temperature of 5000 K after applying small random shifts to the model ('shake' term set to 0.3). The resultant *mF*obs – *dF*calc difference electron density maps are shown in Figure S3.

SUPPLEMENTAL REFERENCES

Adams, P.D., Afonine, P.V., Bunkóczi, G., Chen, V.B., Davis, I.W., Echols, N., Headd, J.J., Hung, L.W., Kapral, G.J., Grosse-Kunstleve, R.W., McCoy, A.J., Moriarty, N.W., Oeffner, R., Read, R.J., Richardson, D.C., Richardson, J.S., Terwilliger, T.C., and Zwart, P.H. (2010) PHENIX: a comprehensive Python-based system for macromolecular structure solution. Acta. Crystallogr. D. Biol. Crystallogr. *66*, 213-21.

Cowtan, K. (2006). The Buccaneer software for automated model building. 1. Tracing protein chains. Acta. Crystallogr. D. Biol. Crystallogr. *62*, 1002-11.

Cowtan, K. (2010). Recent developments in classical density modification. Acta Crystallogr Sect D *66*, 470-8.

Davis, I.W., Leaver-Fay, A., Chen,V.B., Block, J.N., Kapral, G.J., Wang, X., Murray, L.W., Arendall, W.B., Snoeyink, J., Richardson, J.S., and Richardson, D.C. (2007) MolProbity: all-atom contacts and structure validation for proteins and nucleic acids. Nucleic Acids Res. *35*, W375-83.

Doublié, S. (1997). Preparation of selenomethionyl proteins for phase determination. Methods Enzymol. *276*, 523-30.

Emsley, P., and Cowtan, K. (2004). Coot: model-building tools for molecular graphics. Acta. Crystallogr. D. Biol. Chrystallogr. *60*, 2126-32.

Evans, P.R., and Murshudov, G.N. (2013). How good are my data and what is the resolution? Acta. Crystallogr. D. Biol. Crystallogr. *69*, 1204-14.

Holm, L., and Sander, C. (1995). DALI: a network tool for protein structure comparison. Trends Biochem. Sci. *20*, 478-480.

Kabsch W. (2010). XDS. Acta. Crystallogr. D. Biol. Crystallogr. *66*, 125-32.

McCoy, A.J., Grosse-Kunstleve, R.W., Adams, P.D., Winn, M.D., Storoni, L.C., and Read, R.J. (2007). Phaser chrystallographic software. J. Appl. Chrystallogr. *40*, 658-674.

Murshudov, G.N., Vagin, A.A., and Dodson, E.J. (1997). Refinement of macromolecular structures by the maximum-likelihood method. Acta. Crystallogr. D. Biol. Crystallogr. *53*, 240-55.

Painter, J, and Merritt, E.A. (2006). Optimal description of a protein structure in terms of multiple groups undergoing TLS motion. Acta. Crystallogr. D. Biol. Crystallogr. *62*, 439-50.

Read RJ. (1986). Improved Fourier coefficients for maps using phases from partial structures with errors. Acta. Crystallogr. A. *42*, 140-149.

Sheldrick GM. 2008. A short history of SHELX. Acta. Crystallogr. A. *64*, 112-22.

Winter G. (2010). Xia2: an expert system for macromolecular crystallography data reduction. J. Appl. Chrystallogr. *43*, 186-190.
