# Supplementary material for: Substrate-Assisted Catalysis in Polyketide Reduction Proceeds via a Phenolate Intermediate
Source: Cell Chem Biol. 2016 Sep 22;23(9):1091–7. doi: 10.1016/j.chembiol.2016.07.018 (PMC5039031; doi:10.1016/j.chembiol.2016.07.018)
Supplement: Document S2. Article plus Supplemental Information [file mmc2.pdf]

Brief Communication

Cell Chemical Biology

Substrate-Assisted Catalysis in Polyketide Reduction Proceeds via a Phenolate Intermediate

Graphical Abstract

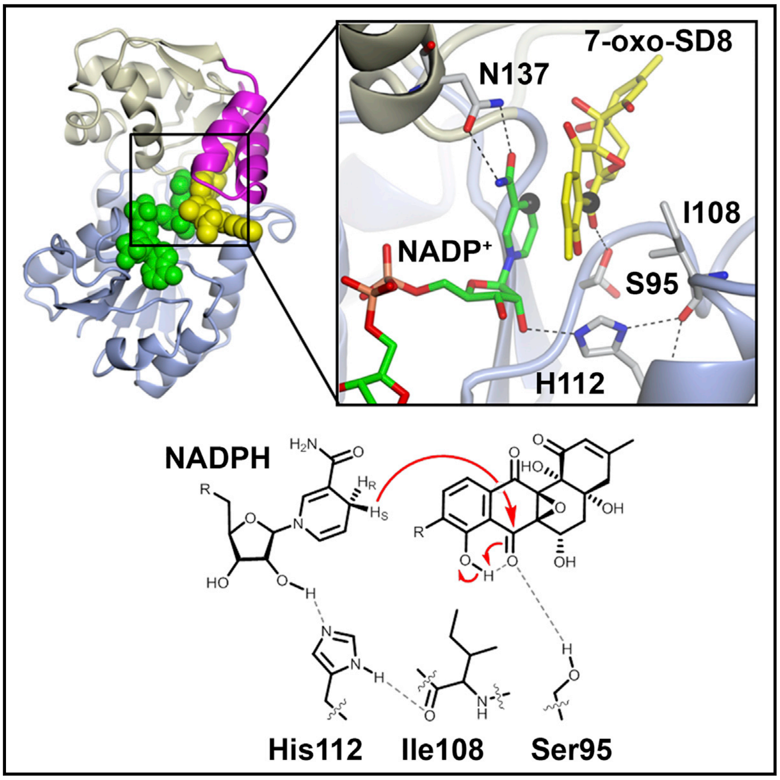

Highlights

- SimC7 catalyzes reduction of the C-7 carbonyl of the angucyclinone of simocyclinone
- SimC7 lacks the conserved catalytic triad characteristic of canonical ketoreductases
- SimC7 reduces the C-7 carbonyl via a substrate-assisted, two-step reaction
- This involves intramolecular transfer of a substrate proton to generate a phenolate

Authors

Martin Schäfer, Clare E.M. Stevenson, Barrie Wilkinson, David M. Lawson, Mark J. Buttner

Correspondence

mark.buttner@jic.ac.uk

In Brief

SimC7 is a polyketide ketoreductase involved in the biosynthesis of the angucyclinone moiety of the DNA gyrase inhibitor simocyclinone. It is structurally distinct from previously characterized ketoreductases, lacking the canonical Ser-Tyr-Lys catalytic triad. Instead, SimC7 catalyzes a substrate-assisted, two-step reaction involving an unusual phenolate intermediate.

Accession Numbers

5L40
5L45
5L3Z
5L4L

Schäfer et al., 2016, Cell Chemical Biology *23*, 1091–1097
September 22, 2016 © 2016 The Author(s). Published by Elsevier Ltd.
http://dx.doi.org/10.1016/j.chembiol.2016.07.018

Please cite this article as: Schäfer et al., Substrate-Assisted Catalysis in Polyketide Reduction Proceeds via a Phenolate Intermediate, Cell Chemical Biology (2016), http://dx.doi.org/10.1016/j.chembiol.2016.07.018

Cell Chemical Biology
Brief Communication

CellPress

Substrate-Assisted Catalysis in Polyketide Reduction Proceeds via a Phenolate Intermediate

Martin Schäfer,[1] Clare E.M. Stevenson,[2] Barrie Wilkinson,[1] David M. Lawson,[2] and Mark J. Buttner[1,3,*]
[1]Department of Molecular Microbiology
[2]Department of Biological Chemistry
John Innes Centre, Norwich Research Park, Norwich NR4 7UH, UK
[3]Lead Contact
*Correspondence: mark.buttner@jic.ac.uk
http://dx.doi.org/10.1016/j.chembiol.2016.07.018

SUMMARY

SimC7 is a polyketide ketoreductase involved in biosynthesis of the angucyclinone moiety of the gyrase inhibitor simocyclinone D8 (SD8). SimC7, which belongs to the short-chain dehydrogenase/reductase (SDR) superfamily, catalyzes reduction of the C-7 carbonyl of the angucyclinone, and the resulting hydroxyl is essential for antibiotic activity. SimC7 shares little sequence similarity with characterized ketoreductases, suggesting it might have a distinct mechanism. To investigate this possibility, we determined the structures of SimC7 alone, with $NADP^+$, and with $NADP^+$ and the substrate 7-oxo-SD8. These structures show that SimC7 is distinct from previously characterized polyketide ketoreductases, lacking the conserved catalytic triad, including the active-site tyrosine that acts as central acid-base catalyst in canonical SDR proteins. Taken together with functional analyses of active-site mutants, our data suggest that SimC7 catalyzes a substrate-assisted, two-step reaction for reduction of the C-7 carbonyl group involving intramolecular transfer of a substrate-derived proton to generate a phenolate intermediate.

INTRODUCTION

Angucyclin(on)es form the largest group of polycyclic aromatic polyketides, many with anticancer and antibacterial activities (Kharel et al., 2012). They share a polyketide-derived tetracyclic benz[*a*]anthracene carbon skeleton, but numerous structures are generated by a range of tailoring reactions, including O- or C-linked deoxysugar glycosylation to form angucyclines. In general, these tailoring enzymes are not well understood. An important challenge, therefore, is to define the step catalyzed by each enzyme and to determine their reaction mechanisms. This knowledge is particularly relevant to the rational engineering of angucyclin(on)e biosynthetic pathways for novel therapeutics.

Simocyclinone D8 (SD8) is a potent DNA gyrase inhibitor isolated from *Streptomyces antibioticus* that consists of an angucyclinone connected to a chlorinated aminocoumarin via a D-olivose deoxysugar and a tetraene diester linker (Figure 1) (Schimana et al., 2000; Edwards et al., 2009; Hearnshaw et al., 2014). SD8 is bifunctional, with the angucyclinone and the aminocoumarin at opposite ends of the molecule binding to two distinct pockets on the DNA binding surface of the GyrA subunit of gyrase (Edwards et al., 2009; Hearnshaw et al., 2014), thereby inhibiting DNA supercoiling at submicromolar concentrations (Edwards et al., 2011). Because gyrase is essential in bacteria but absent from humans, it is an attractive target for antimicrobial drugs, as exemplified by the clinically successful fluoroquinolones (Collin et al., 2011).

SimC7 was originally annotated as a dehydratase and predicted to be involved in the biosynthesis of the tetraene linker of SD8 (Trefzer et al., 2002). However, we recently showed that SimC7 is in fact an NAD(P)H-dependent ketoreductase that catalyzes the reduction of a carbonyl to a hydroxyl group at the C-7 position of the angucyclinone (Schäfer et al., 2015). This enzymatic step is essential for antibiotic activity, converting the almost inactive 7-oxo-simocyclinone D8 (7-oxo-SD8; half maximal inhibitory concentration [IC_{50}] ~50–100 μM) into the potent gyrase inhibitor SD8 (IC_{50} ~0.1–0.6 μM) (Schäfer et al., 2015).

Based on the intermediates produced by *S. antibioticus*, it seems that the biosynthesis of SD8 starts with assembly of the angucyclinone, followed by the attachment of the deoxysugar, then the tetraene linker, and finally the aminocoumarin (i.e., SD8 is assembled from right to left in Figure 1) (Schimana et al., 2001). Therefore, the physiological substrate of SimC7 is most likely a 7-oxo angucyclinone intermediate lacking the attached deoxysugar, tetraene linker, and aminocoumarin, an intermediate that is detectable only in *ΔsimC7* mutants (Schäfer et al., 2015). Despite this, the enzyme readily accepts as a substrate the full-length intermediate 7-oxo-SD8, the major product made by *ΔsimC7* mutants (Schäfer et al., 2015).

The angucyclinone moiety of SD8 is synthesized by a type II polyketide synthase (SimA1-3) and multiple tailoring enzymes (SimA4-13, SimC7) that catalyze cyclization, aromatization, oxidation, and reduction reactions. Several ketoreductases of the short-chain dehydrogenase/reductase (SDR) family that act on angucyclinones or related polyketides have been characterized. The reduction of carbonyl groups at the C-6 and C-9 positions of polyketides has been functionally characterized, and the structures of the corresponding SDR enzymes have elucidated their reaction mechanisms and factors determining their stereoselectivity. The ketoreductases LanV and UrdMred act on the C-6 carbonyl group of angucyclic polyketides from the landomycin and urdamycin pathways (Paananen et al., 2013; Patrikainen

This is an open access article under the CC BY license (http://creativecommons.org/licenses/by/4.0/).

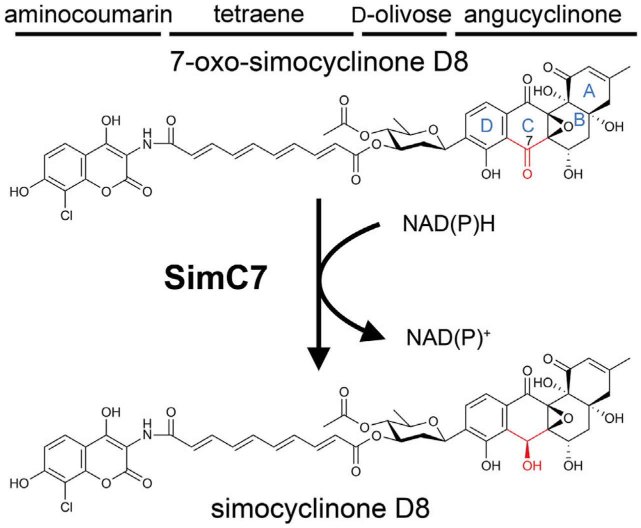

Figure 1. SimC7 Catalyzes the Reduction of 7-oxo-SD8 to Simocyclinone D8

A, B, C, and D denote the four rings of the angucyclinone moiety; the C-7 carbonyl/hydroxyl is highlighted in red.

et al., 2014). In contrast, the ketoreductases ActKR and HedKR act on the C-9 carbonyl group of early intermediates in the actinorhodin and hedamycin polyketide pathways (Javidpour et al., 2011a, 2011b, 2013; Korman et al., 2004, 2008). The LanV, UrdMred, ActKR, and HedKR structures revealed the catalytic Ser-Tyr-Lys triad characteristic of SDR enzymes, where the latter two residues form a YxxxK motif. In these classical SDR proteins, the conserved active-site tyrosine serves as central acid-base catalyst that donates a proton to the substrate. The adjacent lysine residue lowers the pK_a of the tyrosine hydroxyl group and often contributes directly to a proton relay mechanism, and the hydroxyl group of the serine stabilizes and polarizes the carbonyl group of the substrate (Kavanagh et al., 2008).

At the sequence level, SimC7 shares little similarity with any characterized ketoreductase, even with functionally analogous polyketide ketoreductases. The striking differences between the amino acid sequence of SimC7 and those of HedKR, ActKR, LanV, and UrdMred suggested that SimC7 might have a novel catalytic mechanism. To investigate this possibility, we determined the structures of SimC7 alone (apo; 1.6 Å resolution), the binary complex with $NADP^+$ (1.95 Å), and the ternary complex with both $NADP^+$ and 7-oxo-SD8 (1.2 Å) (Tables S1 and S2). Our results reveal that SimC7 is structurally distinct from previously characterized polyketide ketoreductases and, importantly, lacks the canonical SDR Ser-Tyr-Lys catalytic triad (Kavanagh et al., 2008; Kallberg et al., 2010; Persson and Kallberg, 2013). Instead, our data suggest that SimC7 catalyzes a substrate-assisted, two-step reaction for the reduction of the C-7 carbonyl group involving an unusual phenolate intermediate.

RESULTS AND DISCUSSION

Overall Structure of SimC7

SimC7 is made up of two domains, the larger of which is the nucleotide binding domain that adopts a Rossmann fold (Figures 2A, 2B, and S1); the smaller substrate binding domain, characteristic of the so-called extended SDR subfamily (Kavanagh et al., 2008), is mainly α-helical and is largely formed by two insertions in the nucleotide binding domain (between β6 and α6, and between β9 and α10). Notably, the latter insertion contains a "lid" motif consisting of two antiparallel α helices (α8 and α9) that folds over the active site (Figures 2A, 2B, and S1). The substrate binding domain is completed by a short helical segment at the C-terminus of the polypeptide chain.

Overall the apo, binary, and ternary SimC7 structures are very similar (Table S3), with the notable exception of the lid motif, which displays a number of different conformations (Figure 2C). Although the changes are not large (maximum Cα-Cα shift 5.35 Å; Table S3), there is a clear closure of the lid over the bound substrate (Figure 2C), suggesting a role in gating access to the active site and/or substrate capture. Moreover, in the ternary complex the underside of the lid contributes to the tight, highly hydrophobic substrate binding pocket (Figure 3) that provides the necessary environment for catalysis (see below).

Structural Homologs of SimC7

Structures annotated as SDR proteins (PFAM family PF00106) are prevalent in the PDB, with more than 600 entries. To look for structural homologs of SimC7, we carried out a structure-based similarity search using the DALI server (Table S4). Strikingly, characterized angucyclinone ketoreductases ranked very low in the search, the closest match being LanV (Figure S2A), which was the 166th ranked hit after filtering for sequence redundancy (Table S4). Instead, the two most structurally similar proteins to SimC7 were quinone oxidoreductase (QOR2) from *Escherichia coli* (PDB: 2ZCV) (Kim et al., 2008a) (Figure S2A) and triphenylmethane reductase (TMR) from *Citrobacter* sp. KCTC 18061P (PDB: 2VRB) (Kim et al., 2008b). QOR2 and TMR share with SimC7 the ability to reduce substrates with extensively conjugated pi systems but have roles in the detoxification of xenobiotics rather than the biosynthesis of natural products. The majority of the closest structural homologs of known function are involved in sugar biosynthesis, many of them epimerases.

SimC7 and its closest structural homologs all fall into the extended SDR subfamily of proteins, characterized by having two distinct domains that together form a partially occluded active-site pocket at their junction (Figure S2A). In contrast, the other structurally characterized polyketide ketoreductases such as LanV are more distantly related and belong to the classical SDR subfamily (Kavanagh et al., 2008), in which three insertions within the core Rossmann fold motif delineate a more accessible active-site cavity but do not constitute a well-defined substrate binding domain (Figure S2A).

Substrate Binding

In the ternary complex with substrate, determined at 1.2-Å resolution, the angucyclic ring system of 7-oxo-SD8 binds adjacent and parallel to the nicotinamide ring of the cofactor (Figures 2D, 2E, and S3), where it adopts a relatively planar conformation differing from the conformations seen in the DNA gyrase-SD8 and SimR-SD8 complexes, in which the A ring of the angucyclinone in SD8 is tilted upward toward the epoxide (Hearnshaw et al., 2014; Le et al., 2011) (Figure S2B). This planar conformation is most likely enforced by the shape of the very constricted

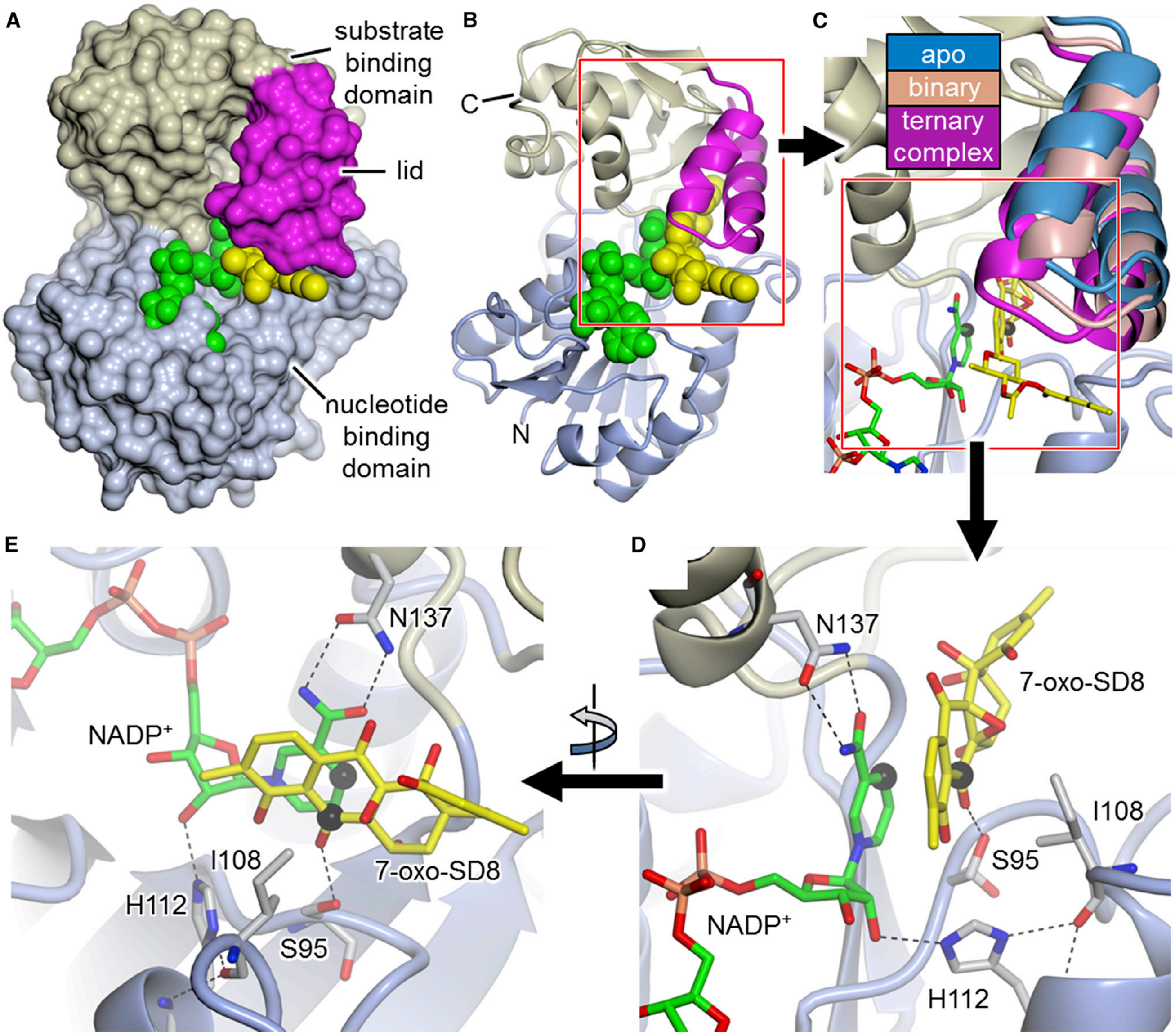

Figure 2. Crystal Structure of SimC7

(A and B) SimC7 displayed as (A) a molecular surface and (B) cartoon representation with the nucleotide binding domain, the substrate binding domain, and the lid motif shown in pale blue, beige, and magenta, respectively. Ligands are represented as van der Waals spheres with the 7-oxo-SD8 substrate shown in yellow and the cofactor shown in green.

(C) Close-up showing conformational changes in the lid between the apo (chain A, form 2; blue), the binary complex (salmon), and the ternary complex (magenta); the core protein structure and ligands shown represent the ternary complex. C-4 of the cofactor and C-7 of the substrate are highlighted by black spheres, showing that C-7 of the substrate is exactly positioned 3 Å from C-4 of the nicotinamide ring, poised for direct hydride transfer.

(D and E) Orthogonal close-ups showing the active site of the ternary complex including the Ser95-Ile108-His112 "catalytic triad" residues, and Asn137, which is important in maintaining the *syn* conformation of the cofactor. For clarity, only the angucyclic polyketide moiety of the substrate is shown. Hydrogen bonds are shown as dashed lines. (E) is also reproduced as a stereo image in Figures S3A and S3B, which also show a difference electron density map calculated from the final model after simulated annealing refinement with the substrate omitted.

See also Figures S1–S4 and Tables S1–S3.

and highly hydrophobic substrate pocket (Figure 3). Within this hydrophobic pocket, 7-oxo-SD8 is bound only by a single direct hydrogen bond between the side chain of Ser95 and the C-7 carbonyl oxygen of the angucyclinone moiety, which may help to position the latter exactly above the C-4 position of the nicotinamide ring, where it is poised for direct hydride transfer (highlighted by black spheres in Figures 2C–2E, 3B, and S3). As mentioned above, the natural substrate for SimC7 is likely to be a 7-oxo angucyclinone intermediate lacking the deoxysugar, the tetraene linker, and the aminocoumarin. Consistent with this, only the angucyclinone moiety is buried in the active site of SimC7. Roughly half of the tetraene linker is visible in the electron

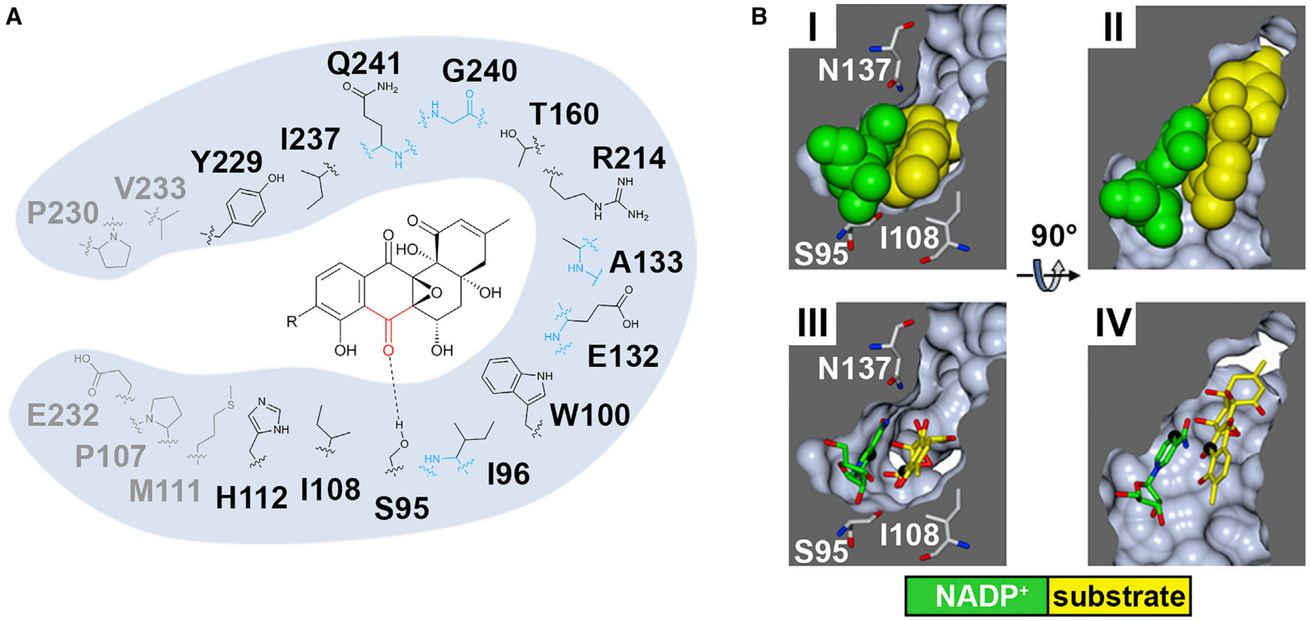

Figure 3. SimC7 Has a Very Hydrophobic and Constricted Substrate Binding Pocket

(A) The substrate is bound by only one direct hydrogen bond between the C-7 carbonyl group (red) in the angucyclinone moiety and the side-chain hydroxyl of Ser95. This interaction may assist in positioning the substrate and facilitating the reaction. Interacting residues are shown in black (side-chain interactions) or blue (backbone interactions). The hydrophobic residues shown in gray line the entrance to the substrate pocket but do not interact directly with the bound angucyclinone. Note that one face of the pocket is formed by the cofactor itself (not shown). In the natural SimC7 substrate R=H, in the substrate used here R includes the deoxysugar, tetraene linker, and the aminocoumarin.

(B) Orthogonal cross-sections through the active-site pocket, revealing how tightly cofactor (green) and substrate (yellow) are bound. For clarity, only the nicotinamide ribosyl moiety of the cofactor and the polyketide moiety of the substrate are shown. In (I) and (III) the view corresponds roughly to that shown in Figure 2D, whereas (II) and (IV) show the view from above relative to Figure 2D. In the lower panels, C-4 of the cofactor and C-7 of the substrate are highlighted by black spheres, showing that C-7 of the substrate is exactly positioned 3 Å from C-4 of the nicotinamide ring, poised for hydride transfer.

See also Figures S1–S3.

density, projecting away from the protein surface, and the aminocoumarin ring is not resolved at all (Figure S3).

A Novel Catalytic Mechanism for a Polyketide Ketoreductase

SimC7 lacks the Ser-Tyr-Lys catalytic triad characteristic of canonical SDR proteins (Figure 4 and Table S4). While the serine is conserved (Ser95), the other two residues (i.e., the YxxxK motif) are not, being instead replaced by Ile108 and His112, respectively. This Ser-Ile-His triad is unlike any described for the five subfamilies of SDRs defined by Kavanagh et al. (2008). Particularly surprising is the absence of the tyrosine residue that acts as the acid-base catalyst in the classical SDR mechanism (Figure 4A). Inspection of the structure of the ternary complex shows that none of the five tyrosine residues in SimC7 is sufficiently close to C-7 of the angucyclinone ring system of the substrate to play a direct role in catalysis. Furthermore, the structure also shows that there is no alternative residue that could act as an acid-base catalyst. Consequently, SimC7 must perform ketoreduction of 7-oxo-SD8 via a novel mechanism.

Based on the structure of the ternary complex of the enzyme with $NADP^+$ and 7-oxo-SD8, we propose a simple two-step mechanism for SimC7 that does not depend on catalytic residues in the protein, but rather takes advantage of the specific properties of the substrate itself, and is thus a novel example of substrate-assisted catalysis (Dall'Acqua and Carter, 2000). In the first step, the hydrophobic environment of the substrate binding pocket and the juxtaposition of the quinone-like C ring and the phenyl-like D ring of the angucyclinone favor the formation of an intramolecular hydrogen bond between the proton on the C-8 hydroxyl group and the oxygen of the neighboring C-7 carbonyl group (Figure 4B). This enhances the polarization of the latter such that the electrophilicity of C-7 is increased, making it a good acceptor for direct hydride transfer from the 4-*pro-S* position of the nicotinamide ring. Crucially, the hydride donor and acceptor carbon atoms are only 3.0 Å apart in the crystal structure. The C-7 hydroxyl group is then formed by internal proton transfer from the neighboring C-8 hydroxyl group, generating a phenolate intermediate in which the negative charge on the C-8 oxygen atom is stabilized by the aromatic D ring. In the second step of the reaction, the phenolate intermediate leaves the substrate binding pocket and the proton required to reinstate the C-8 hydroxyl group is recovered by abstraction from bulk water, which is not possible within the confines of the active site (Figure 4B). Exchange of the negatively charged reaction intermediate is most likely accelerated by repulsion from the hydrophobic active-site cavity. Finally, the direct hydride attack from below the angucyclic polyketide unambiguously explains the 7*S* stereochemistry of simocyclinones. In support of this proposed mechanism, molecular modeling predicts the existence of the key intramolecular hydrogen bond between the C-8 hydroxyl group and the C-7 carbonyl group of 7-oxo-SD8, and an

Please cite this article as: Schäfer et al., Substrate-Assisted Catalysis in Polyketide Reduction Proceeds via a Phenolate Intermediate, Cell Chemical Biology (2016), http://dx.doi.org/10.1016/j.chembiol.2016.07.018

A

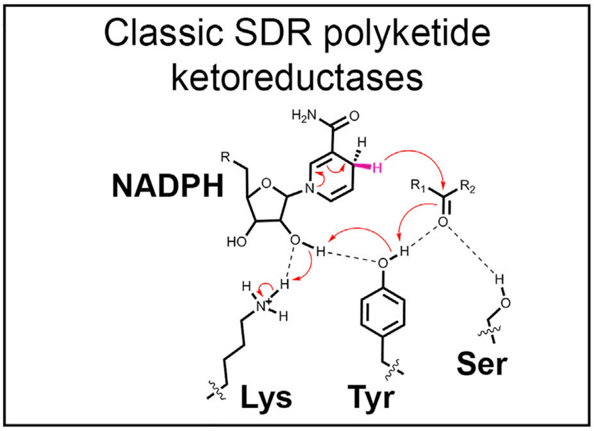

C

Mutant	Conversion (%)	Specific activity (nmol min^{-1} mg^{-1})
wildtype	100.0 ± 7.3	13.9 ± 1.0
S95A	86.1 ± 7.1	11.9 ± 1.0
I108A	91.5 ± 13.8	12.7 ± 1.9
I108D	1.2 ± 0.8	0.2 ± 0.1
H112A	12.5 ± 3.6	1.7 ± 0.5
H112N	5.8 ± 2.5	0.8 ± 0.4
H112Q	1.0 ± 1.4	0.1 ± 0.2

B

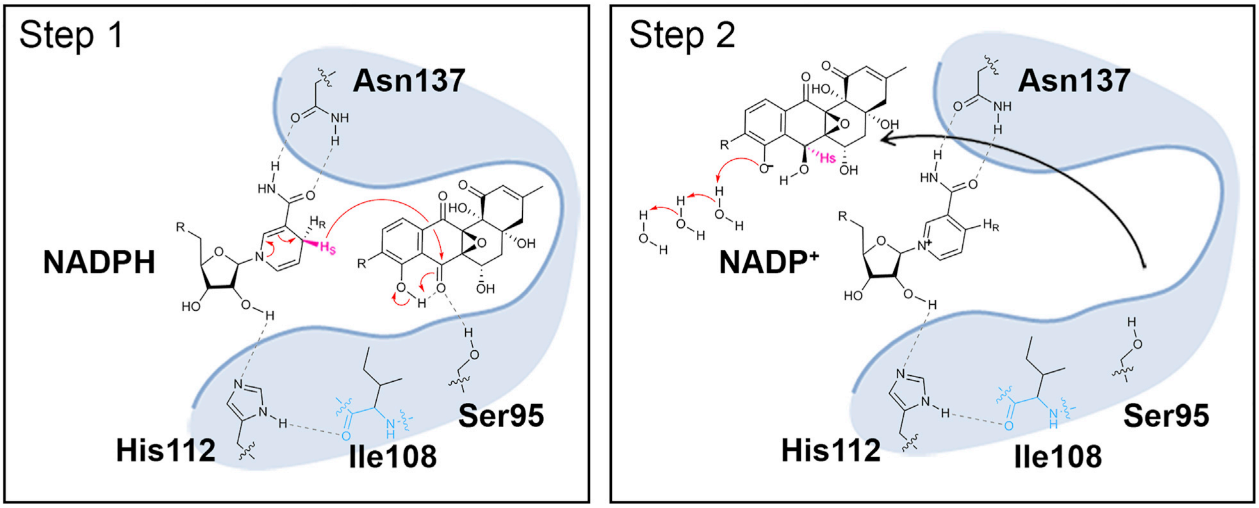

Figure 4. Canonical SDR Ketoreduction and the Novel SimC7 Reaction Mechanism

(A) In canonical SDR proteins the conserved active-site tyrosine serves as central acid-base catalyst that donates a proton to the substrate. The adjacent lysine residue lowers the pK_a of the tyrosine hydroxyl group and often contributes directly to the proton relay mechanism; the hydroxyl group of the serine stabilizes and polarizes the carbonyl group of the substrate.

(B) SimC7 has an atypical catalytic triad comprised of Ser95, Ile108, and His112. In the first step of the proposed SimC7 mechanism, the C-7 carbonyl group of the substrate (7-oxo-SD8) is reduced by transfer of the 4-pro-*S* hydride of the cofactor onto the C-7 carbon of the substrate. This transfer from below the C ring results in the characteristic 7-*S* stereochemistry of SD8. Ketoreduction at position C-7 is completed by an intramolecular proton transfer from the neighboring C-8 hydroxyl group of the angucyclinone; the resultant negative charge on the latter is stabilized by the adjacent aromatic ring system (ring D in Figure 1). In the second step, the C-8 phenolate intermediate regains a proton from bulk water after leaving the substrate binding pocket. In the natural SimC7 substrate R=H, in the substrate used here R includes the deoxysugar, tetraene linker, and the aminocoumarin. Note that there are no water molecules in the active-site pocket that could contribute to the reaction mechanism. In the ternary complex, the nearest water to O-7 of the angucyclic polyketide is ~5.5 Å away, and the nearest water to O-8 is ~4.9 Å away. Due to steric constraints within the pocket, neither could approach the substrate oxygen atoms without either a repositioning of the substrate or a conformational change in the protein.

(C) Enzymatic activities of SimC7 active-site mutants. Standard errors are indicated for three independent experiments.

See also Figures S3 and S4; Table S4.

equivalent intramolecular hydrogen bond is observed in the small-molecule crystal structure of panglimycin, a closely related polyketide (Fotso et al., 2008). Furthermore, molecular modeling also predicts that the C-8 hydroxyl group will have the most acidic and exchangeable proton in the angucyclic polyketide, with an estimated pK_a of 6.9–7.7, and therefore could readily transfer to the neighboring C-7 oxygen at the end of step 1. Indeed, there is no other possible proton donor (neither protein nor water derived) sufficiently close to O-7 to perform this role.

Based on their structures (Figure S4A), there are four other angucyclinones in which a SimC7-like mechanism might generate a C-7 hydroxyl group: panglimycin, elmycin, grisemycin, and kiamycin (Fotso et al., 2008; Xie et al., 2012, 2016). However, the biosynthetic gene clusters for these molecules have yet to be reported, and it is not known whether a SimC7-like enzyme is involved in their synthesis. Most other angucyclin(on)es have a carbonyl group at C-7.

Mutagenesis of the SimC7 Active Site

To investigate the potential roles of the SimC7 "catalytic triad" residues in the proposed reaction mechanism, we mutagenized Ser95, Ile108, and His112 (Figure 4B). In the wild-type enzyme,

the hydroxyl group of Ser95 could aid catalysis by helping to bind and correctly orient the substrate, and by providing additional polarization to the C-7 carbonyl group via a hydrogen bond, the latter role being consistent with the function proposed for the structurally equivalent Ser/Thr residues in the classical SDR mechanism (Figure 4A) (Kavanagh et al., 2008; Kallberg et al., 2010; Persson and Kallberg, 2013). However, neither effect would appear to be crucial for activity, as a S95A mutant showed 86% substrate conversion relative to the wild-type (Figure 4C). Therefore, SimC7 must be able to orient 7-oxo-SD8 for catalysis without the necessity for the hydrogen bond from Ser95, and this likely arises because the active-site cavity of the binary complex closely matches the shape of the substrate (Figure 3B).

Ile108 contributes to the hydrophobic surface that forms one side of the active-site cavity and has a nonspecific role in helping to trap the angucyclinone group against the nicotinamide ring of the cofactor (Figure 3B). Unsurprisingly, an I108A substitution had almost no effect, whereas changing it to aspartate (I108D) abolished activity (Figure 4C). In the latter case, the introduction of a negative charge would interfere with the hydrophobic environment and could disrupt the intramolecular hydrogen bond in the substrate, both being necessary for catalysis.

Finally, mutation of His112 (H112A, H112N, and H112Q) strongly reduced or abolished enzyme activity (Figure 4C), consistent with its key role in binding and positioning the cofactor via a hydrogen bond to the 2′-hydroxyl of the nicotinamide ribosyl moiety (Figures 2D, 2E, and 4B).

SIGNIFICANCE

The sequence of SimC7 is distinct from previously characterized polyketide ketoreductases, and the structural data reported here suggest that it catalyzes a novel substrate-assisted, two-step reaction for the reduction of the C-7 carbonyl group. This mechanism involves the intramolecular transfer of a substrate-derived proton to generate a phenolate intermediate, negating the need for proton transfer from a canonical SDR active-site tyrosine. Like SimC7 (Ser-Ile-His), the two closest structural homologs, TMR (Tyr-Leu-His) and QOR2 (Leu-Leu-His), also have unusual active-site triads. Thus SimC7, TMR, and QOR2 share an I/LxxxH motif and have substrates with extensively conjugated pi systems. No enzyme-substrate complex crystal structures have been described for TMR or QOR2 and no firm proposals exist for their catalytic mechanisms, but their substrate structures and the data provided here suggest they are also likely to employ noncanonical mechanisms. Our data therefore point to members of the extended SDR sub-family having I/LxxxH active site motifs as a source of new biochemistry.

EXPERIMENTAL PROCEDURES

For a full explanation of the experimental protocols, see Supplemental Experimental Procedures.

Protein Overexpression and Purification

Point mutants of *simC7* were generated by PCR-based site-directed mutagenesis. All constructs were verified by sequencing. Proteins were expressed in *E. coli* as N-terminally His-tagged fusions, purified by nickel-affinity chromatography and assayed by high-performance liquid chromatography as described previously (Schäfer et al., 2015). The structural integrity of purified proteins was verified using circular dichroism.

Protein Crystallization and Structure Determination

SimC7 was labeled with selenomethionine (SeMet) by metabolic inhibition, and crystals of both native and SeMet proteins were grown by vapor diffusion (crystals of binary and ternary complexes were obtained by cocrystallization). Crystals were harvested and flash-cooled in liquid nitrogen. The native crystals did not require further cryoprotection, while the SeMet-labeled crystals were cryoprotected by supplementing the crystallization solution with 25% (v/v) glycerol. All X-ray data were collected at the Diamond Light Source. For de novo structure determination, a single-wavelength anomalous dispersion dataset was collected at the Se *K* X-ray absorption edge for an SeMet-labeled SimC7 crystal. Native and SeMet data were combined to solve the structure of SimC7 in complex with $NADP^+$. All other structures were determined by molecular replacement using the latter as a template.

ACCESSION NUMBERS

The accession numbers for the SimC7 structures reported in this paper are PDB: 5L40 (apo form 1), 5L45 (apo form 2), 5L3Z (binary complex), and 5L4L (ternary complex).

SUPPLEMENTAL INFORMATION

Supplemental Information includes Supplemental Experimental Procedures, four figures, and four tables and can be found with this article online at http://dx.doi.org/10.1016/j.chembiol.2016.07.018.

AUTHOR CONTRIBUTIONS

All authors designed the experiments. M.S. and C.E.M.S. performed the experiments. All authors analyzed the data. M.S., D.M.L., and M.J.B. wrote the paper, and all authors made revisions.

ACKNOWLEDGMENTS

We thank Hans-Peter Fiedler for providing simocyclinone D8, and the staff of the Diamond Light Source (Oxford, UK) for access to beamlines I03, I04, and I04-1 under proposal MX9475. This work was funded by a BBSRC studentship to M.S., by BBSRC grant BB/I002197/1 to M.J.B. and D.M.L., and by BBSRC Grant BB/J004561/1 to the John Innes Centre.

Received: June 9, 2016
Revised: July 17, 2016
Accepted: July 26, 2016
Published: September 8, 2016

REFERENCES

Collin, F., Karkare, S., and Maxwell, A. (2011). Exploiting bacterial DNA gyrase as a drug target: current state and perspectives. Appl. Microbiol. Biotechnol. *92*, 479–497.

Dall'Acqua, W., and Carter, P. (2000). Substrate-assisted catalysis: molecular basis and biological significance. Protein Sci. *9*, 1–9.

Edwards, M.J., Flatman, R.H., Mitchenall, L.A., Stevenson, C.E.M., Le, T.B.K., Clarke, T.A., McKay, A.R., Fiedler, H.-P., Buttner, M.J., Lawson, D.M., and Maxwell, A. (2009). A crystal structure of the bifunctional antibiotic, simocyclinone D8, bound to DNA gyrase. Science *326*, 1415–1418.

Edwards, M.J., Williams, M.A., Maxwell, A., and McKay, A.R. (2011). Mass spectrometry reveals that the antibiotic simocyclinone D8 binds to DNA gyrase in a "bent-over" conformation: evidence of positive cooperativity in binding. Biochemistry *50*, 3432–3440.

Fotso, S., Mahmud, T., Zabriskie, T.M., Santosa, D.A., Sulastri, and Proteau, P.J. (2008). Angucyclinones from an Indonesian *Streptomyces* sp. J. Nat. Prod. *71*, 61–65.

Hearnshaw, S.J., Edwards, M.J., Stevenson, C.E., Lawson, D.M., and Maxwell, A. (2014). A new crystal structure of the bifunctional antibiotic simocyclinone D8 bound to DNA gyrase gives fresh insight into the mechanism of inhibition. J. Mol. Biol. *426*, 2023–2033.

Javidpour, P., Das, A., Khosla, C., and Tsai, S.C. (2011a). Structural and biochemical studies of the hedamycin type II polyketide ketoreductase (HedKR): molecular basis of stereo- and regiospecificities. Biochemistry *50*, 7426–7439.

Javidpour, P., Korman, T.P., Shakya, G., and Tsai, S.C. (2011b). Structural and biochemical analyses of regio- and stereospecificities observed in a type II polyketide ketoreductase. Biochemistry *50*, 4638–4649.

Javidpour, P., Bruegger, J., Srithahan, S., Korman, T.P., Crump, M.P., Crosby, J., Burkart, M.D., and Tsai, S.C. (2013). The determinants of activity and specificity in actinorhodin type II polyketide ketoreductase. Chem. Biol. *20*, 1225–1234.

Kallberg, Y., Oppermann, U., and Persson, B. (2010). Classification of the short-chain dehydrogenase/reductase superfamily using hidden Markov models. FEBS J. *277*, 2375–2386.

Kavanagh, K.L., Jörnvall, H., Persson, B., and Oppermann, U. (2008). The SDR superfamily: functional and structural diversity within a family of metabolic and regulatory enzymes. Cell. Mol. Life Sci. *65*, 3895–3906.

Kharel, M.K., Pahari, P., Shepherd, M.D., Tibrewal, N., Nybo, S.E., Shaaban, K.A., and Rohr, J. (2012). Angucyclines: biosynthesis, mode-of-action, new natural products, and synthesis. Nat. Prod. Rep. *29*, 264–325.

Kim, I.K., Yim, H.S., Kim, M.K., Kim, D.W., Kim, Y.M., Cha, S.S., and Kang, S.O. (2008a). Crystal structure of a new type of NADPH-dependent quinone oxidoreductase (QOR2) from *Escherichia coli*. J. Mol. Biol. *379*, 372–384.

Kim, M.H., Kim, Y., Park, H.J., Lee, J.S., Kwak, S.N., Jung, W.H., Lee, S.G., Kim, D., Lee, Y.C., and Oh, T.K. (2008b). Structural insight into bioremediation of triphenylmethane dyes by *Citrobacter* sp. triphenylmethane reductase. J. Biol. Chem. *283*, 31981–31990.

Korman, T.P., Hill, J.A., Vu, T.N., and Tsai, S.C. (2004). Structural analysis of actinorhodin polyketide ketoreductase: cofactor binding and substrate specificity. Biochemistry *43*, 14529–14538.

Korman, T.P., Tan, Y.H., Wong, J., Luo, R., and Tsai, S.C. (2008). Inhibition kinetics and emodin cocrystal structure of a type II polyketide ketoreductase. Biochemistry *47*, 1837–1847.

Le, T.B.K., Stevenson, C.E.M., Fiedler, H.-P., Maxwell, A., Lawson, D.M., and Buttner, M.J. (2011). Structures of the TetR-like simocyclinone efflux pump repressor, SimR, and the mechanism of ligand-mediated derepression. J. Mol. Biol. *408*, 40–56.

Paananen, P., Patrikainen, P., Kallio, P., Mäntsälä, P., Niemi, J., Niiranen, L., and Metsä-Ketelä, M. (2013). Structural and functional analysis of angucycline C-6 ketoreductase LanV involved in landomycin biosynthesis. Biochemistry *52*, 5304–5314.

Patrikainen, P., Niiranen, L., Thapa, K., Paananen, P., Tähtinen, P., Mäntsälä, P., Niemi, J., and Metsä-Ketelä, M. (2014). Structure-based engineering of angucyclinone 6-ketoreductases. Chem. Biol. *21*, 1381–1391.

Persson, B., and Kallberg, Y. (2013). Classification and nomenclature of the superfamily of short-chain dehydrogenases/reductases (SDRs). Chem. Biol. Interact. *202*, 111–115.

Schäfer, M., Le, T.B.K., Hearnshaw, S.J., Maxwell, A., Challis, G.L., Wilkinson, B., and Buttner, M.J. (2015). SimC7 is a novel NAD(P)H-dependent ketoreductase essential for the antibiotic activity of the DNA gyrase inhibitor simocyclinone. J. Mol. Biol. *427*, 2192–2204.

Schimana, J., Fiedler, H.P., Groth, I., Sussmuth, R., Beil, W., Walker, M., and Zeeck, A. (2000). Simocyclinones, novel cytostatic angucyclinone antibiotics produced by *Streptomyces antibioticus* Tu 6040. I. Taxonomy, fermentation, isolation and biological activities. J. Antibiot. (Tokyo) *53*, 779–787.

Schimana, J., Walker, M., Zeeck, A., and Fiedler, H.-P. (2001). Simocyclinones: diversity of metabolites is dependent on fermentation conditions. J. Ind. Microbiol. Biotechnol. *27*, 144–148.

Trefzer, A., Pelzer, S., Schimana, J., Stockert, S., Bihlmaier, C., Fiedler, H.-P., Welzel, K., Vente, A., and Bechthold, A. (2002). Biosynthetic gene cluster of simocyclinone, a natural multihybrid antibiotic. Antimicrob. Agents Chemother. *46*, 1174–1182.

Xie, Z., Liu, B., Wang, H., Yang, S., Zhang, H., Wang, Y., Ji, N., Qin, S., and Laatsch, H. (2012). Kiamycin, a unique cytotoxic angucyclinone derivative from a marine *Streptomyces* sp. Mar. Drugs *10*, 551–558.

Xie, Z., Zhou, L., Guo, L., Yang, X., Qu, G., Wu, C., and Zhang, S. (2016). Grisemycin, a bridged angucyclinone with a methylsulfinyl moiety from a marine-derived *Streptomyces* sp. Org. Lett. *18*, 1402–1405.

Cell Chemical Biology, Volume *23*

Supplemental Information

Substrate-Assisted Catalysis in Polyketide Reduction Proceeds via a Phenolate Intermediate

Martin Schäfer, Clare E.M. Stevenson, Barrie Wilkinson, David M. Lawson, and Mark J. Buttner

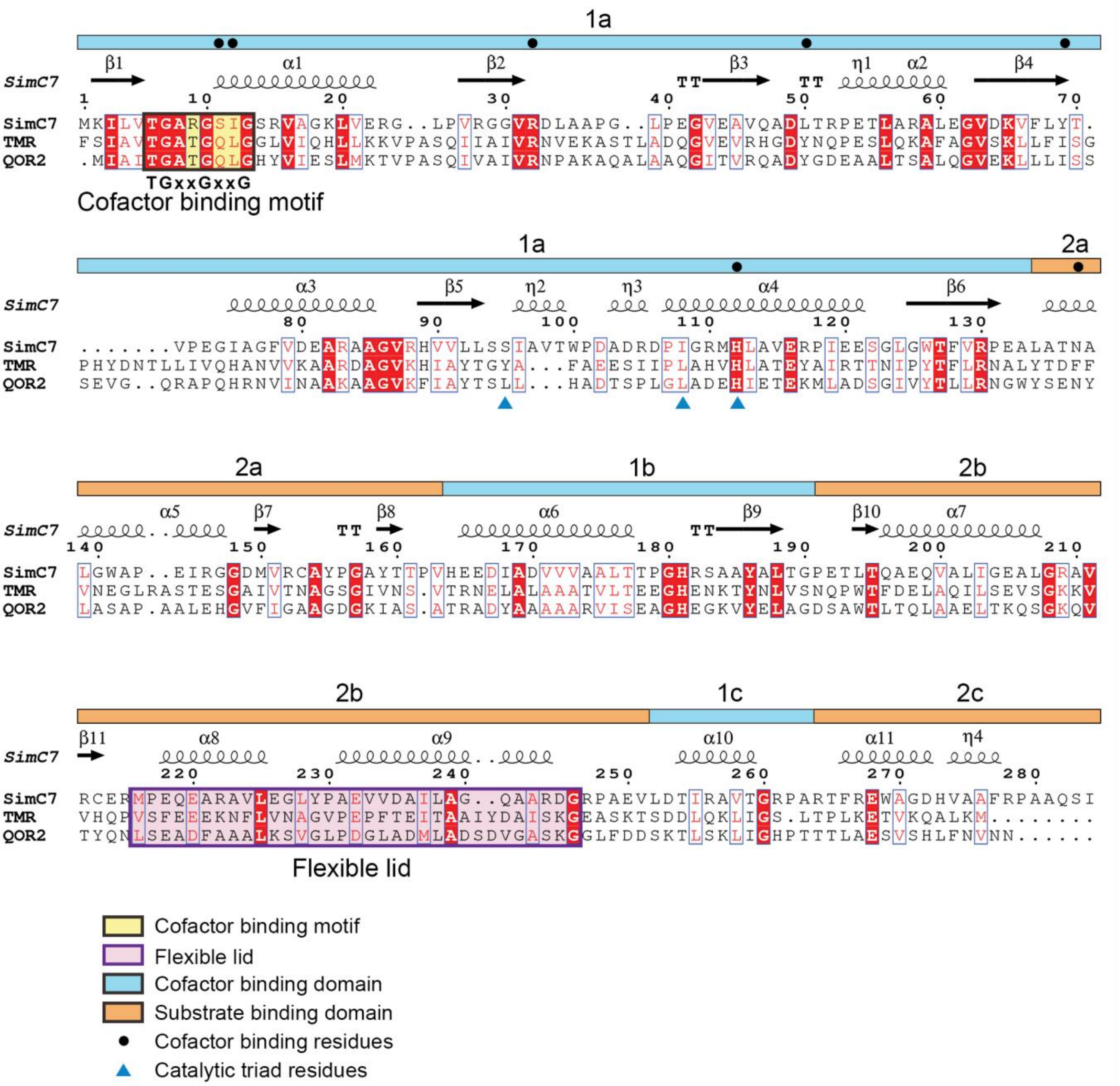

Figure S1, related to Figure 2. Structure-based sequence alignment of SimC7 with triphenylmethane reductase (TMR) from *Citrobacter* sp. and quinone oxidoreductase (QOR2) from *E. coli*. Conserved residues are shown in white on a red background and similar residues are shown in red font. Secondary structure for SimC7 is shown above the alignment (α for α-helix and β for β-strand, TT for β-turn and η for 3_{10} helix). The bars above the alignment indicate the cofactor binding domain (1a-c in blue) and the substrate binding domain (2a-c in orange) with black dots indicating residues that interact with the $NADP^+$ cofactor. The conserved N-terminal cofactor-binding motif (TGxxGxxG, yellow with black frame) and the flexible lid (Met216 to Gly246, pink frame) are highlighted. Blue triangles indicate the positions equivalent to the active site triad residues in canonical SDR proteins.

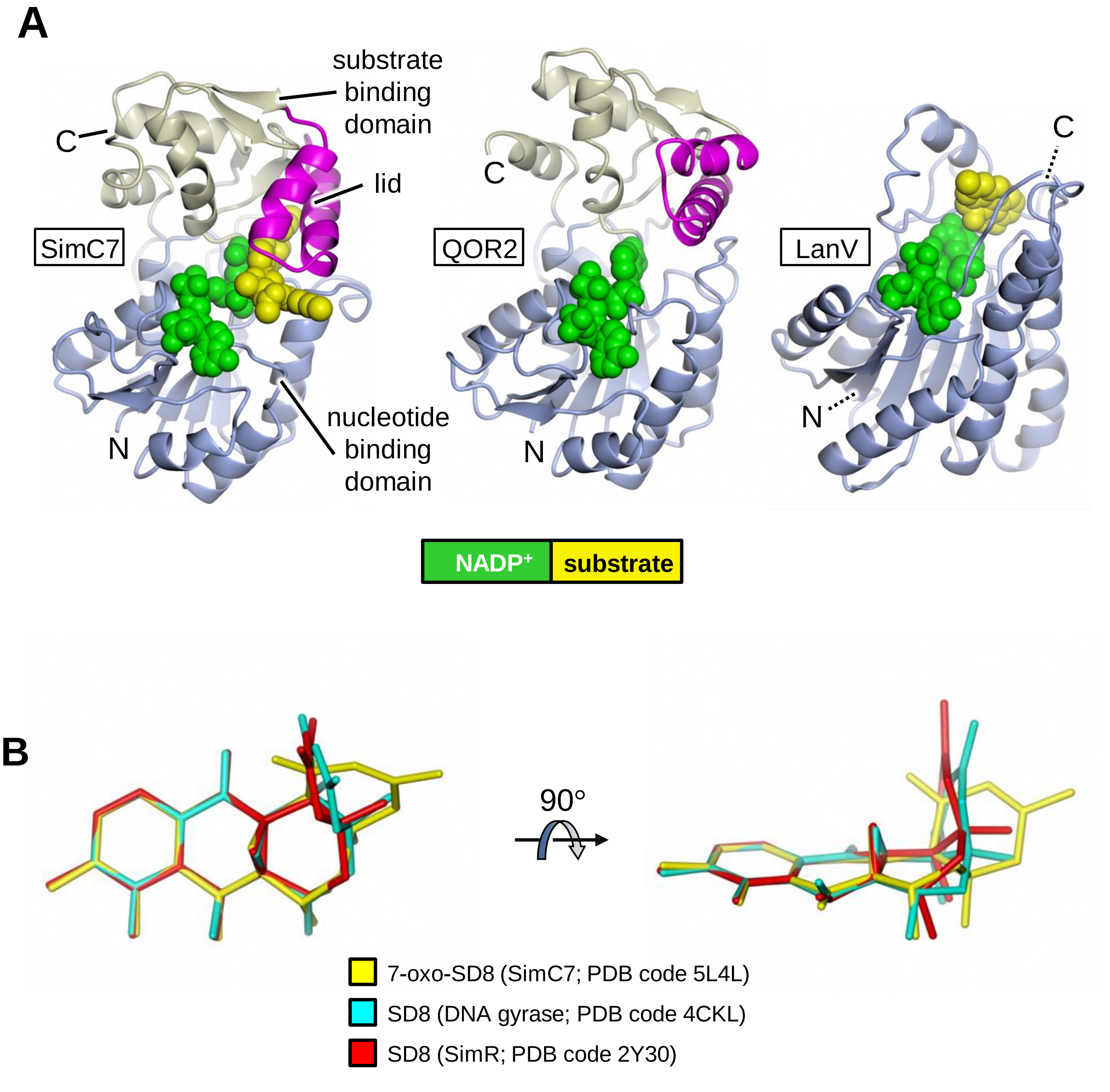

Figure S2, related to Figure 2. (A) Comparison of the ternary complexes of SimC7 and LanV, and the binary complex of QOR2. Structures are depicted in cartoon representation, where pale blue, beige and magenta colouration indicate the nucleotide binding domain, the substrate binding domain and the lid motif, respectively (N.B. there is no distinct substrate binding domain or lid motif in LanV). The ligands are shown as van der Waals spheres, with the $NADP^+$ cofactor shown in green and the substrate (where present) shown in yellow. See Table S4 for a more extensive comparison of SimC7 structural homologues. (B) Comparison of the 7-oxo-SD8 conformation in the ternary complex of SimC7 with SD8 conformations observed previously in other complexes. Orthogonal views showing a least-squares superposition of the angucyclic moiety of 7-oxo-SD8 (based on the 6 carbon atoms of ring D) upon SD8 from the complex with GyrA55 and from the complex with SimR.

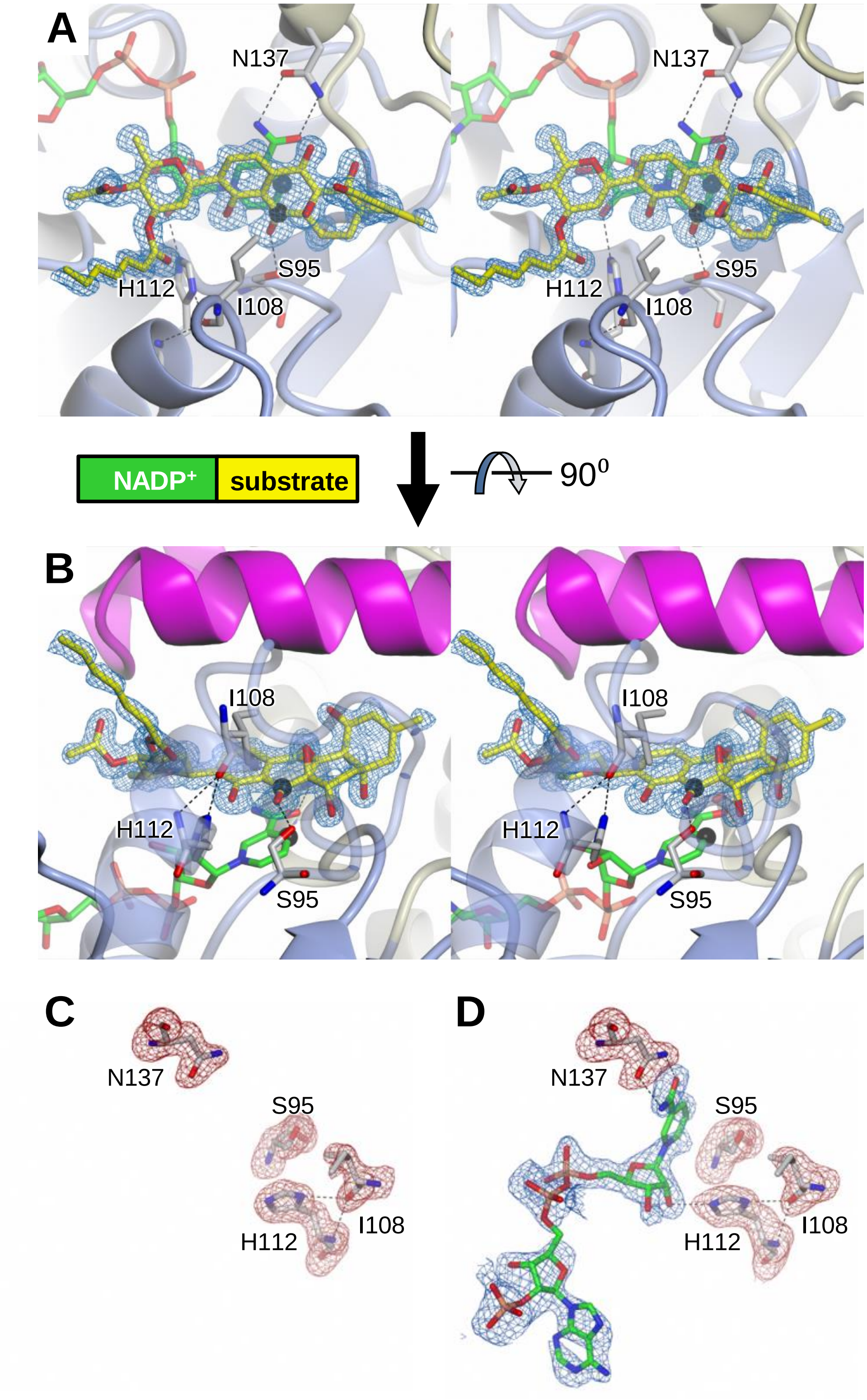

A
N137
H112
S95
I108
N137
H112
S95
I108
NADP+
substrate
90⁰
B
I108
H112
S95
I108
H112
S95
C
N137
S95
H112
I108
D
N137
S95
H112
I108

Figure S3, related to Figure 2. Simulated annealing omit electron density maps. Parts (A) and (B) show orthogonal stereo views of the active site of the SimC7 ternary complex. Ligands are depicted in stick representation, where green indicates carbon atoms of the cofactor and yellow, carbons of the substrate; C-4 of the cofactor and C-7 of the substrate are specifically highlighted by small black spheres. Superposed upon the substrate in blue is a simulated annealing omit map calculated at 1.2 Å resolution (see Extended Experimental Procedures). Also shown are the catalytic triad residues, as well as Asn137, which is important in maintaining the *syn* conformation of the cofactor. The view in part (A) is equivalent to that in Fig. 2E (again the lid motif has been removed for clarity). In part (B), the protein backbone encompassing the β5 - α4 loop and bearing the catalytic triad has been shown in transparent mode as it would otherwise obscure the detail of the active site; the lid is in magenta. Parts (C) and (D) show omit maps (red density) for just the active site residues in Apo form 1 (1.6 Å resolution) and the binary complex (1.95 Å resolution), respectively. In the latter panel a separate omit map is also shown (blue density), which was calculated from the final model lacking only the cofactor. The view in parts (C) and (D) is roughly equivalent to that used in Fig. 2D. All omit maps were contoured at ~3.0 σ.

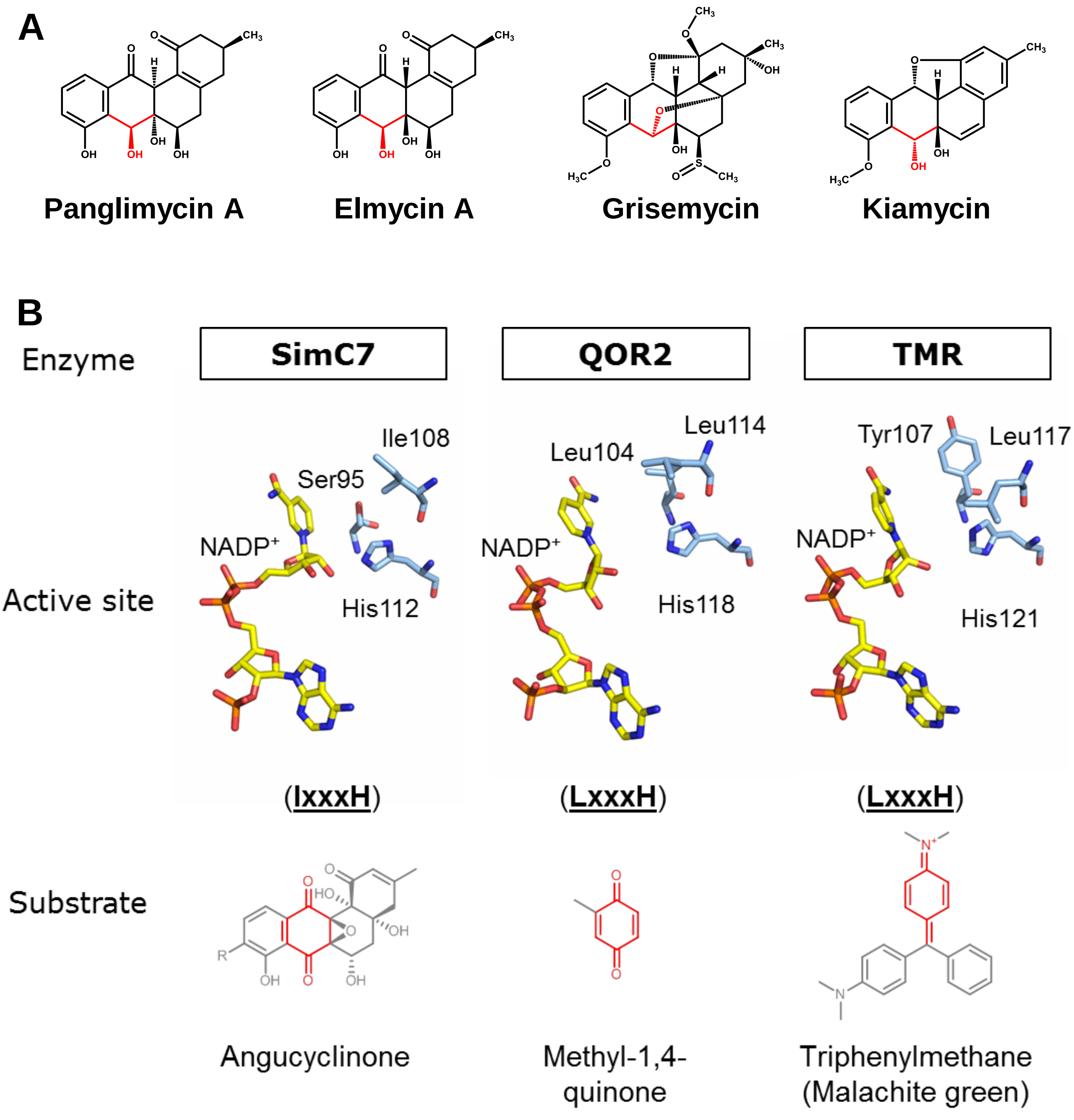

Figure S4, related to Figure 4. (A) Structures of angucyclinones in which a SimC7-like mechanism might generate a C-7 hydroxyl group. Panglimycin, elmycin, grisemycin and kiamycin are all produced by *Streptomyces* strains but their biosynthetic gene clusters have not been reported. The unusual intramolecular ether bridge between C-4a and C-7 in grisemycin is most likely formed from a precursor having hydroxyl groups at both positions. (B) Variations in the active site triad and substrates between SimC7, QOR2 and TMR. Arrangement of the cofactor (yellow) and the protein residues (blue) that constitute the active site triad in SimC7, quinone oxidoreductase (QOR2, PDB accession number 2ZCV) from *E. coli*, and triphenylmethane reductase (TMR, PDB accession number 2JL1) from *Citrobacter* sp. The substrate of each enzyme is shown below. The natural substrate of TMR is not known.

Table S1, related to Figure 2. X-ray data collection and processing

Data set	Binary SeMet	Binary native	Apo form 1	Apo form 2	$NADP^+$ plus 7-oxo ternary complex
Beamline	I03	I03	I04-1	I04-1	I04
Wavelength (Å)	0.9796	1.0052	0.9173	0.9173	0.9795
Detector	Pilatus 6M	Pilatus 6M	Pilatus 6M	Pilatus 6M	Pilatus 6M
Resolution range (Å)[a]	51.48 – 2.05 (2.10 – 2.05)	50.74 – 1.95 (2.00 – 1.95)	53.40 – 1.60 (1.64 – 1.60)	54.88 – 1.90 (1.95 – 1.90)	47.52 – 1.20 (1.23 – 1.20)
Space Group	$P4_12_12$	$P4_12_12$	$P4_12_12$	C2	$P2_12_12_1$
a, b, c (Å)	51.5, 51.5, 213.2	52.2, 52.2, 214.0	52.4, 52.4, 213.6	107.0, 64.8, 91.5	51.9, 53.7, 102.3
α, β, γ (°)	90.0, 90.0, 90.0	90.0, 90.0, 90.0	90.0, 90.0, 90.0	90.0, 105.5, 90.0	90.0, 90.0, 90.0
Total observations[a]	457751 (25343)	563166 (40131)	435288 (32092)	336053 (23051)	1159483 (81348)
Unique reflections[a]	19033 (1335)	22650 (1625)	40514 (2936)	47271 (3433)	90009 (6590)
Multiplicity[a]	24.1 (19.0)	24.9 (24.7)	10.7 (10.9)	7.1 (6.7)	12.9 (12.3)
Mean $I/\sigma(I)$[a]	16.5 (1.3)	18.4 (2.2)	20.0 (1.5)	13.6 (1.0)	17.5 (2.1)
Completeness (%)[a]	99.8 (99.7)	99.9 (99.8)	99.9 (99.2)	98.5 (96.1)	100.0 (99.5)
R_{merge}[a,b]	0.122 (2.745)	0.118 (1.845)	0.066 (1.814)	0.087 (1.990)	0.070 (1.208)
R_{meas}[a,c]	0.125 (2.820)	0.120 (1.883)	0.069 (1.903)	0.094 (2.157)	0.073 (1.249)
$CC_{½}$[a,d]	0.999 (0.673)	0.999 (0.819)	1.000 (0.701)	0.999 (0.543)	0.999 (0.760)
Wilson B value ($Å^2$)	45.3	34.0	21.6	34.2	11.9

[a] Values for the outer resolution shell are given in parentheses.
[b] $R_{merge} = \sum_{hkl} \sum_i |I_i(hkl) - \langle I(hkl)\rangle| / \sum_{hkl} \sum_i I_i(hkl)$.
[c] $R_{meas} = \sum_{hkl} [N/(N-1)]^{1/2} \times \sum_i |I_i(hkl) - \langle I(hkl)\rangle| / \sum_{hkl} \sum_i I_i(hkl)$, where $I_i(hkl)$ is the ith observation of reflection hkl, $\langle I(hkl)\rangle$ is the weighted average intensity for all observations i of reflection hkl and N is the number of observations of reflection hkl.
[d] $CC_{½}$ is the correlation coefficient between symmetry-related intensities taken from random halves of the dataset.

Table S2, related to Figure 2. Refinement of X-ray structures

Data set	Binary native	Apo form 1	Apo form 2	NADP[+] plus 7-oxo ternary complex
Resolution range (Å)[a]	50.74 – 1.95 (2.00 – 1.95)	53.40 – 1.60 (1.64 – 1.60)	54.88 – 1.90 (1.95 – 1.90)	47.52 – 1.20 (1.23 – 1.20)
Reflections: working/free[b]	21479/1171	38504/2010	44913/2357	85532/4476
Final R_{work}[a,c]	0.182 (0.282)	0.189 (0.297)	0.195 (0.342)	0.126 (0.218)
Final R_{free}[a,c]	0.217 (0.316)	0.218 (0.303)	0.229 (0.348)	0.149 (0.230)
Cruickshank DPI (Å)[d]	0.139	0.087	0.150	0.032
R.m.s. bond deviations (Å)	0.010	0.010	0.009	0.010
R.m.s. angle deviations (°)	1.40	1.38	1.29	1.52
No. of protein residues (ranges)	280 (1 to 280)	280 (1 to 280)	A chain:278 (2-279); B chain 281 (-1 to 279)[*]	278 (2-279)
No. of heterogen residues: cofactor/ 7-oxo/water/other	1/0/104/0	0/0/167/0	0/0/202/2	1/1/324/1
Mean *B*-factors: protein/cofactor/ 7-oxo/water/other/overall (Å^2)	47/46[#]/-/46/-/47	35/-/-/39/-/35	50/-/-/49/58/50	17/11/21/32/28/19
Ramachandran plot: favoured/allowed/ disallowed (%)[e]	98.6/1.4/0.0	99.3/0.7/0.0	98.4/1.2/0.4	99.1/0.9/0.0
PDB accession code	5L3Z	5L40	5L45	5L4L

[a] Values for the outer resolution shell are given in parentheses.
[b] The data set was split into "working" and "free" sets consisting of 95 and 5% of the data, respectively. The free set was not used for refinement.
[c] The R-factors R_{work} and R_{free} are calculated as follows: $R = \sum(| F_{obs} - F_{calc} |)/\sum| F_{obs} |$, where F_{obs} and F_{calc} are the observed and calculated structure factor amplitudes, respectively.
[d] Diffraction precision indicator based on R_{free} based as calculated by *REFMAC5* (Murshudov et al., 1997).
[e] As calculated using *MOLPROBITY* (Davis et al., 2007).
[*] Two residues were visible for the N-terminal His-tag in the B-chain. Since the numbering scheme was based on the wild-type sequence, these residues were labelled as “-1” and “0”.
[#] Cofactor was refined with occupancy of 0.7.

Table S3 related to Figure 2. Comparison of SimC7 structures

Overall r.m.s. deviations (Å) [maximum Cα shift (Å)/ corresponding residue]	Apo form 1	Apo form 2: A chain	Apo form 2: B chain	Binary complex	Ternary complex
Apo form 1	0.00	1.13 [4.18/P37]* [3.82/A231]	0.58 [1.71/P230]	0.18‡ [0.40/R222]	0.84 [3.67/G227]
Apo form 2: A chain		0.00	0.88 [3.15/E232]	1.10 [3.92/A231]	1.43 [5.35/G227]
Apo form 2: B chain			0.00	0.58 [1.65/P230]	0.98 [4.20/G227]
Binary complex				0.00	0.83 [3.67/G227]
Ternary complex					0.00

Pairwise superpositions of all protein structures determined herein; R.m.s. deviations were determined by LSQKAB [Kabsch, 1976]. For Apo form 2, the two molecules in the ASU were treated as separate models.

*With the exception of this comparison, all the others showed the biggest shift in the lid region (residues 216-246 inclusive). Here the biggest shift was due to a *trans/cis* isomerisation of Pro37. The second largest shift (in the lid) is also shown.

‡ N.B. Apo form 1 and the binary complex are isomorphous.

Table S4, related to Figure 2. Selected structural homologues of SimC7

Protein	Source	Biological unit[a]	PDB code[b]	Ligand bound[c]	Resolution (Å)	DALI output					"Catalytic" triad	Reference
						Rank[d]	Z-score	R.m.s.d. (Å)	aligned residues	Identity (%)		
SimC7	*Streptomyces antibioticus* Tü6040	Monomer	5L4L	NADP$^{\pm}$ & 7oxo-SD8	1.20	-	-	0.00	278	100	Ser95, Ile108, His112	This work
QOR2	*Escherichia coli*	Monomer	2ZCV	NADP^{+}	1.60	1	27.4	3.1	266	23	Leu104, Leu114, His118	Kim *et al*., 2008
TMR	*Citrobacter* sp. KCTC 18061P	Dimer	2JL1	NADP^{+}	1.96	2	27.0	3.2	268	22	Tyr107, Leu117, His121	Kim *et al*., 2008
HSCARG	*Homo sapiens*	Monomer/ Dimer	2EXX	NADP$^{\pm}$	2.40	4	24.8	3.1	263	22	Leu114, His129, Lys133	Zheng *et al*., 2007
UDP-hexose 4-epimerase	*Thermotoga maritima*	Dimer	4ZRM	NADP$^{\pm}$ & UDP-Glc	2.00	6	22.9	2.5	247	20	Thr117, Tyr143, Lys147	Shin *et al*., 2015
Cinnamoyl-CoA reductase	*Petunia hybrid*	(Monomer)	4R1S	NADP$^{\pm}$	1.60	8	22.4	3.0	246	22	Ser123, Tyr157, Lys161	Pan *et al*., 2014
LanV	*Streptomyces cyanogenus*	Dimer	4KWI	NADP$^{\pm}$ & Lando	2.00	166	14.3	3.0	173	22	Ser147, Tyr160, Lys164	Paananen *et al*., 2013
UrdMred	*Streptomyces fradiae*	(Tetramer)	4OSP	NADP$^{\pm}$ & rabelomycin	2.25	167	14.3	3.2	177	20	Ser147, Tyr160, Lys164	Patrikainen *et al*., 2104
HedKR	*Streptomyces griseoruber*	(Tetramer)	3SJU	NADP$^{\pm}$	2.40	N/A	15.5	4.0	185	19	Ser142, Tyr155, Lys159	Javidpour *et al*., 2011
ActKR	*Streptomyces coelicolor*	Tetramer	2RHC	NADP$^{\pm}$ & Emodin	2.10	N/A	15.1	3.9	185	18	Ser144, Tyr157, Lys161	Korman *et al*., 2008

[a] Experimentally determined (e.g. by size exclusion chromatography), unless shown in brackets, in which case it was inferred from the crystal structure alone. For HSCARG, the oligomeric state (as judged by dynamic light scattering) was dependent on the NADP^{+} concentration.
[b] Results of a DALI search (http://ekhidna.biocenter.helsinki.fi/dali_server) (Holm and Sander, 1995) using the SimC7 ternary complex structure as the template. The hits were filtered for redundancy; where a relevant ligand bound structure exists for a particular enzyme, this is the one that is shown.
[c] Lando = 11-deoxy-6-oxylandomycinone. Where the cofactor is underlined, this indicates that the nicotinamde ring adopts the *syn* configuration like SimC7; otherwise the configuration is *anti*.
[d] Ranking of the DALI hit (after redundancy filtering at 90% sequence identity). For some classes of enzymes, e.g. the sugar epimerases, only the top hit is shown. The lower ranking hits LanV and UrdMred were also chosen as they were relevant to the discussion. For the same reason, ActKR and HedKR were included, despite not being picked up by a blind DALI search.

SUPPLEMENTAL EXPERIMENTAL PROCEDURES

Site-directed mutagenesis of *simC7*

The PCR-based Q5® site-directed mutagenesis kit [New England Biolabs] was used to generate *simC7* point mutants, using plasmid pET15b-NB-C7 as the template. Pairs of oligonucleotides were designed to amplify linearized plasmid DNA with one oligonucleotide containing the desired mutation. The template plasmid was degraded with *Dpn*I and the linear PCR product was self-ligated in the presence of kinase and DNA ligase before transformation into *E. coli* DH5α. Mutations were verified by DNA sequencing.

Oligonucleotides used. Mutant sites are underlined and in uppercase.

**Oligonucleotide**	**Mutation**	**Sequence (5'-3')**
S95A-F	S95A	tgctctccGCCatcgccgtgacctggc
S95AT-R	S95A	gcaccacatgccggaccc
I108A-F	I108A	gacccgGCGggccggatgcacctc
I108D-F	I108D	gacccgGATggccggatgcacctc
I108A-R	I108A/D	ccggtccgcgtccggccaggtc
H112A-F	H112A	ggatgGCCctcgccgtcga
H112N-F	H112N	ggatgAACctcgccgtcgagcg
H112Q-F	H112Q	ggatgCAGctcgccgtcgagcg
H112R-R	H112A/N/Q	ggccgatcgggtcccgg

Protein overexpression and purification

N-terminally His_6-tagged SimC7 and its mutant derivatives were expressed from pET15b-NB-C7 in *E. coli* Rosetta(DE3) pLysS cells (Schäfer *et al.*, 2015). The recombinant protein had 20 additional amino acids at the N-terminus compared to the native sequence (MGSSHHHHHHSSGLVPRGSH) and a molecular weight of 32,235 Da. We established from a number of expression and purification trials that the presence of sodium chloride had a tendency to induce aggregation of protein derived from this construct. For this reason, sodium chloride was either excluded or kept at low concentrations in the buffers used thereafter. 10 mL overnight culture was used to inoculate 1 L of Luria-Bertani medium without sodium chloride containing 40 mg carbenicillin and 10 mg chloramphenicol. The culture was grown at 37 °C to OD_{600} ~0.3, cooled to 18 °C, and protein expression was induced by addition of IPTG to a final concentration of 0.5 mM. The culture was incubated for 16 h shaking at 18 °C. Harvested cells were resuspended in lysis buffer [20 mM Na-HEPES (pH 8.0), 40 mM NaCl, 10% (v/v) glycerol, 10 mM imidazole] containing complete EDTA-free protease inhibitor cocktail (Roche), and lysed in a cell disruptor (three cycles with 16,000 psi pressure). Cell debris was removed by centrifugation at 40,000 *g* for 30 min, the supernatant was mixed with 1 mL of Ni-NTP-Agarose beads (Qiagen), and the resin was packed into a 5-mL Polypropylene column (Qiagen) equilibrated with wash buffer [20 mM Na-HEPES (pH 8.0), 40 mM NaCl, 10% (v/v) glycerol, 10 mM imidazole]. The column was washed with 700 mL wash buffer and SimC7 was eluted in 10 mL elution buffer [20 mM Na-HEPES (pH 8.0), 40 mM NaCl, 20% (v/v) glycerol, and 300 mM imidazole]. Protein samples were analysed by SDS-PAGE and immediately exchanged into storage buffer [20 mM Na-HEPES (pH 8.0), 10% (v/v) glycerol, 5 mM (w/v) TCEP] and concentrated to 4 mg/mL using a Vivaspin 15 buffer exchange column (Satorius Stedim Biotech, Germany). The CD spectra of all the mutant SimC7 proteins were collected (see below) and found to be unchanged relative to that of the wild-type enzyme.

For *de novo* structure determination, SimC7 was labelled with selenomethionine (SeMet) by metabolic inhibition (Doublie, 1997). Cells were grown in 2x 0.5 L M9 minimal medium at 37°C to OD_{600} ~0.1, amino acids were added (100mg/L each of threonine, lysine and phenylalanine; 50 mg/L each of leucine, isoleucine and valine; 60 mg/L SeMet), and the cultures were further incubated for 90 min. Protein overexpression was induced with 0.5 mM IPTG overnight at 18°C. Labelled protein was purified as described above.

Circular Dichroism

Circular dichroism (CD) spectroscopy experiments were performed using a Chirascan-Plus CD spectrophotometer (Applied Photophysics, Surrey, UK). Concentrated proteins in storage buffer were buffer exchanged against 20 mM potassium phosphate buffer (pH 7.2) with 5% (v/v) glycerol and diluted to 0.2 mg/mL (6.25 µM). CD analysis was carried out at 20 °C using a quartz glass cuvette with a 0.5 mm path length. CD spectra were averaged from four scans collected between 190 nm and 260 nm wavelength, using a bandwidth of 2.0 nm, a step size of 0.5 nm, and one time point per second.

SimC7 ketoreductase assays

SimC7 variants were assayed by UV/vis-HPLC using 500 nM SimC7, 0.3 mM NADH and 0.2 mM 7-oxo-SD8, as described previously (Schäfer *et al*., 2015). Purified SD8 and 7-oxo-SD8 were used as standards. Substrate conversion was determined by quantification of the peak area for the reaction product SD8 based on a serial dilution of SD8 prepared in methanol (0.25-300 µM). Samples (30 µL) were separated on a reverse-phase HPLC column (Phenomenex Gemini-NX 3u C18 110A, 150 mm × 4.6 mm) using a linear gradient of 70-95% (v/v) methanol against 0.1% (v/v) formic acid over 10 min, followed by 95% (v/v) methanol for 5 min at a flow rate of 1 mL/min. Assays were performed in triplicate using the average of two technical replicates for each data point.

Protein crystallization and cryoprotection

All crystallization experiments were performed with N-terminally His-tagged SimC7 at a concentration of approximately 4 mg/mL and at a temperature of 20°C. Screening was conducted by sitting-drop vapor diffusion in MRC 96-well crystallization plates (Molecular Dimensions) with a mixture of 0.3 µl well solution (from both commercial and in-house screens) and 0.3 µl protein solution using an OryxNano crystallization robot (Douglas Instruments). Promising conditions were optimised manually in 24-well hanging-drop vapor diffusion format using XRL plates (Molecular Dimensions) with drops consisting of 1 µl protein and 1 µl precipitant. Tetragonal crystals of the binary complex with $NADP^+$ were obtained from 16% (w/v) PEG-8000 and 20% (v/v) glycerol (native SimC7) or 1.3 M DL-malic acid (pH 7) supplemented with a final concentration of 1% (v/v) DMSO and 2.5 mM EDTA in the drop (SeMet-labelled SimC7). The native crystals did not require further cryoprotection, while the SeMet crystals were cryoprotected by supplementing the crystallization solution with 25% (v/v) glycerol. Crystals of *apo*-SimC7 that were isomorphous with those of the binary complex (apo form 1) grew from 25% (w/v) PEG-6000, 20% (v/v) glycerol in 0.1 M MES pH 6.5, while monoclinic *apo*-SimC7 crystals (apo form 2) were obtained from 16% (w/v) PEG-8000, 18% (v/v) glycerol with 10 mM dithiothreitol (DTT) added to the drop; neither form required further cryoprotection. 7-oxo-SD8 was isolated from *S. antibioticus* Tü6040 as described previously (Schäfer et al., 2015), and was dissolved in DMSO to a concentration of 50 mM prior to mixing with protein. The cofactor $NADP^+$ (Sigma) was dissolved in water to a concentration of 100 mM without adjusting the pH, which was crucial for obtaining crystals. SimC7 was first mixed with $NADP^+$ and then with 7-oxo-SD8, giving a final concentration of 0.12 mM (3.8 mg/mL) protein, 1.5 mM of 7-oxo-SD8, and 3.5 mM of $NADP^+$. The final concentration of DMSO did not exceed 3% (v/v). Orthorhombic crystals of the ternary complex of SimC7 bound to $NADP^+$ and 7-oxo-SD8 grew from 20-25% (w/v) PEG-8000, 20-25% (v/v) glycerol in 0.1 M sodium acetate pH 4.8, and did not require further cryoprotection.

X-ray data collection, structure determination and refinement

Crystals were harvested and flash-cooled in liquid nitrogen using LithoLoops (Molecular Dimensions). The mounted crystals were stored in Unipuck cassettes (MiTeGen) prior to transport to the Diamond Light Source (Oxfordshire, UK), where they were transferred robotically to the goniostat on either beamline I03, I04 or I04-1 and maintained at -173 °C with a Cryojet cryocooler (Oxford Instruments). X-ray diffraction data were recorded using a Pilatus 6M hybrid photon counting detector (Dectris), then integrated using XDS (Kabsch, 2010), and scaled and merged using AIMLESS (Evans and Murshudov, 2013) via the XIA2 expert system (Winter, 2010); the resultant data collection statistics are summarized in Table S1.

Native data from the binary complex with $NADP^+$ were collected to 1.95 Å and processed in space group $P4_12_12$ with an estimated solvent content of 49.3%, based on one copy of the protein chain in the ASU. A SeMet-labelled SimC7 crystal was used to collect a single-wavelength anomalous dispersion data set at the selenium *K* X-ray absorption edge (wavelength 0.9796 Å). The data were processed to 2.05 Å resolution in space group $P4_12_12$ and were isomorphous with the native set. Experimental phases were determined by analyzing the SeMet and native data using the SHELX suite (Sheldrick, 2008). SHELXD located 4 selenium sites and, after phasing with SHELXE (which revealed that $P4_12_12$ was the correct enantiomorph), the figure-of-merit was 0.589 to 1.95 Å resolution. These phases were improved by density modification with PARROT (Cowtan, 2010) and used as input to automated building with BUCCANEER (Cowtan, 2006), which was able to fit 233 residues (82% of the native sequence). After combining phases calculated from this model with the original experimental phases using SIGMAA (Read, 1986), PARROT and BUCCANEER were re-run. This time 277 residues (98% of the native sequence) were fitted giving R_{work} and R_{free} values of 0.263 and 0.298, respectively, at 1.95 Å resolution. The model of the binary complex was completed by several iterations of manual rebuilding in COOT (Emsley and Cowtan, 2004) and restrained refinement in REFMAC5 (Murshudov et al., 1997) using isotropic thermal parameters and TLS group definitions obtained from the TLSMD server (Painter and Merritt, 2006). The statistics of the final model are reported in Table S2.

A dataset was collected to 1.60 Å resolution from apo-SimC7 crystallized in the same form as the binary complex. This was used to generate the model of apo form 1 starting from the model of the binary complex from which the coordinates of the cofactor had been removed. Model building and refinement were completed as above. Crystals of apo form 2 yielded a dataset to 1.90 Å resolution after processing in space group C2, with an estimated solvent content of 48.3% based on two copies of the protein in the ASU. The structure was solved by molecular replacement with PHASER

(McCoy et al., 2007) using the protein component of binary complex structure as the template, and then refined as above.

Data for the ternary complex of SimC7 with $NADP^+$ and 7-oxo-SD8 were processed to 1.20 Å resolution in space group $P2_12_12_1$, giving an estimated solvent content of 45.3% based on one copy of the protein in the ASU. The latter was located using PHASER with the same template as before. For this structure it was possible to use anisotropic thermal parameter refinement in REFMAC5.

Model geometries were validated with MOLPROBITY (Davis et al., 2007) before submission to the Protein Data Bank. The statistics of all final models are summarized in Table S2.

A simulated annealing omit procedure was used to give an unbiased representation of the electron density for specific parts of the structures. For these, the part in question (e.g. the substrate in the ternary complex) was deleted from the coordinates of the final model. The resultant PDB file was used as input to simulated annealing refinement with PHENIX (Adams et al., 2010) from a starting temperature of 5000 K after applying small random shifts to the model (‘shake’ term set to 0.3). The resultant mFobs – dFcalc difference electron density maps are shown in Figure S3.

SUPPLEMENTAL REFERENCES

Adams, P.D., Afonine, P.V., Bunkóczi, G., Chen, V.B., Davis, I.W., Echols, N., Headd, J.J., Hung, L.W., Kapral, G.J., Grosse-Kunstleve, R.W., McCoy, A.J., Moriarty, N.W., Oeffner, R., Read, R.J., Richardson, D.C., Richardson, J.S., Terwilliger, T.C., and Zwart, P.H. (2010) PHENIX: a comprehensive Python-based system for macromolecular structure solution. Acta. Crystallogr. D. Biol. Crystallogr. *66*, 213-21.

Cowtan, K. (2006). The Buccaneer software for automated model building. 1. Tracing protein chains. Acta. Crystallogr. D. Biol. Crystallogr. *62*, 1002-11.

Cowtan, K. (2010). Recent developments in classical density modification. Acta Crystallogr Sect D *66*, 470-8.

Davis, I.W., Leaver-Fay, A., Chen,V.B., Block, J.N., Kapral, G.J., Wang, X., Murray, L.W., Arendall, W.B., Snoeyink, J., Richardson, J.S., and Richardson, D.C. (2007) MolProbity: all-atom contacts and structure validation for proteins and nucleic acids. Nucleic Acids Res. *35*, W375-83.

Doublié, S. (1997). Preparation of selenomethionyl proteins for phase determination. Methods Enzymol. *276*, 523-30.

Emsley, P., and Cowtan, K. (2004). Coot: model-building tools for molecular graphics. Acta. Crystallogr. D. Biol. Chrystallogr. *60*, 2126-32.

Evans, P.R., and Murshudov, G.N. (2013). How good are my data and what is the resolution? Acta. Crystallogr. D. Biol. Crystallogr. *69*, 1204-14.

Holm, L., and Sander, C. (1995). DALI: a network tool for protein structure comparison. Trends Biochem. Sci. *20*, 478-480.

Kabsch W. (2010). XDS. Acta. Crystallogr. D. Biol. Crystallogr. *66*, 125-32.

McCoy, A.J., Grosse-Kunstleve, R.W., Adams, P.D., Winn, M.D., Storoni, L.C., and Read, R.J. (2007). Phaser chrystallographic software. J. Appl. Chrystallogr. *40*, 658-674.

Murshudov, G.N., Vagin, A.A., and Dodson, E.J. (1997). Refinement of macromolecular structures by the maximum-likelihood method. Acta. Crystallogr. D. Biol. Crystallogr. *53*, 240-55.

Painter, J, and Merritt, E.A. (2006). Optimal description of a protein structure in terms of multiple groups undergoing TLS motion. Acta. Crystallogr. D. Biol. Crystallogr. *62*, 439-50.

Read RJ. (1986). Improved Fourier coefficients for maps using phases from partial structures with errors. Acta. Crystallogr. A. *42*, 140-149.

Sheldrick GM. 2008. A short history of SHELX. Acta. Crystallogr. A. *64*, 112-22.

Winter G. (2010). Xia2: an expert system for macromolecular crystallography data reduction. J. Appl. Chrystallogr. *43*, 186-190.
